# K-Ras(V12) differentially affects the three Akt isoforms in lung and pancreatic carcinoma cells and upregulates E-cadherin and NCAM via Akt3

**DOI:** 10.1186/s12964-024-01484-2

**Published:** 2024-01-30

**Authors:** Rebekka Geißert, Angela Lammert, Stefanie Wirth, Rabea Hönig, Dirk Lohfink, Monika Unger, Denis Pek, Konstantin Schlüter, Theresa Scheftschik, Daniel J. Smit, Manfred Jücker, Andre Menke, Klaudia Giehl

**Affiliations:** 1 Signal Transduction of Cellular Motility, Internal Medicine IV, Science Unit for Basic and Clinical Medicine, Justus Liebig University Giessen, Aulweg 128, D-35392, Giessen, Germany; 2 Molecular Oncology of Solid Tumors, Internal Medicine IV, Justus Liebig University Giessen, Aulweg 128, D-35392, Giessen, Germany; 3 Institute of Pharmacology and Toxicology, University of Ulm, D-89069 Ulm, Germany; 4 Institute of Biochemistry and Signal Transduction, University Medical Center Hamburg-Eppendorf, D-20246 Hamburg, Germany

**Keywords:** K-Ras, PI3-kinase, PKB/Akt isoforms, Akt3, E-cadherin, NCAM, Carcinoma cell migration, Cell-cell adhesion, Ras oncogene

## Abstract

K-Ras is the most frequently mutated Ras variant in pancreatic, colon and non-small cell lung adenocarcinoma. Activating mutations in K-Ras result in increased amounts of active Ras-GTP and subsequently a hyperactivation of effector proteins and downstream signaling pathways. Here, we demonstrate that oncogenic K-Ras(V12) regulates tumor cell migration by activating the phosphatidylinositol 3-kinases (PI3-K)/Akt pathway and induces the expression of E-cadherin and neural cell adhesion molecule (NCAM) by upregulation of Akt3. In vitro interaction and co-precipitation assays identified PI3-Kα as a bona fide effector of active K-Ras4B but not of H-Ras or N-Ras, resulting in enhanced Akt phosphorylation. Moreover, K-Ras(V12)-induced PI3-K/Akt activation enhanced migration in all analyzed cell lines. Interestingly, Western blot analyses with Akt isoform-specific antibodies as well as qPCR studies revealed, that the amount and the activity of Akt3 was markedly increased whereas the amount of Akt1 and Akt2 was downregulated in EGFP-K-Ras(V12)-expressing cell clones. To investigate the functional role of each Akt isoform and a possible crosstalk of the isoforms in more detail, each isoform was stably depleted in PANC-1 pancreatic and H23 lung carcinoma cells. Akt3, the least expressed Akt isoform in most cell lines, is especially upregulated and active in Akt2-depleted cells. Since expression of EGFP-K-Ras(V12) reduced E-cadherin-mediated cell-cell adhesion by induction of polysialylated NCAM, Akt3 was analyzed as regulator of E-cadherin and NCAM. Western blot analyses revealed pronounced reduction of E-cadherin and NCAM in the Akt3-kd cells, whereas Akt1 and Akt2 depletion upregulated E-cadherin, especially in H23 lung carcinoma cells. In summary, we identified oncogenic K-Ras4B as a key regulator of PI3-Kα-Akt signaling and Akt3 as a crucial regulator of K-Ras4B-induced modulation of E-cadherin and NCAM expression and localization.

**Supplementary Information:**

The online version contains supplementary material available at 10.1186/s12964-024-01484-2.

## Background

Monomeric Ras GTPases are prime molecular switches of signaling pathways regulating many aspects of physiological and pathophysiological cell behavior, including cell proliferation, survival, apoptosis, differentiation, transformation, and tumor cell migration and invasion. The three ubiquitously expressed *RAS* genes (*HRAS, KRAS, and NRAS*) encode for four Ras isoforms (H-Ras, N-Ras, K-Ras4A and K-Ras4B), which are collectively expressed in each cell [1]. Ras isoforms share more than 90% sequence homology but strongly differ in their last 23/24 amino acids at the C-terminus, designated as the hypervariable region (HVR). K-Ras4A is less abundant and far less studied than K-Ras4B, thus the term ‘K-Ras’ mostly refers to K-Ras4B [2]. Mutations in the three *RAS* genes emerge in approximately 30% of all human tumors and K-Ras is the most frequently mutated variant in carcinomas, with approximately 90% in ductal pancreatic adenocarcinoma (PDAC), 50% in colon carcinoma and 30% in non-small cell lung cancer (NSCLC). Mutations in K-Ras are highly concentrated in codon 12, 13, and 61 resulting in an increased exchange rate from inactive Ras-GDP to active Ras-GTP and subsequently a hyperactivation of effector proteins and downstream signaling pathways [3–5]. The best-characterized effectors of Ras are Raf kinases [6] and phosphatidylinositol 3-kinases (PI3-K) [7] contributing to two signaling pathways believed to play a pivotal role in transformation and tumorigenesis triggered by oncogenic Ras: the Raf/MEK/ERK pathway and the PI3-K/Akt pathway.

The PI3-K/Akt (protein kinase-B) pathway is an essential regulator of cell proliferation and survival. Dysregulation in this pathway is highly involved in cancer initiation, progression, and recurrence in all types of cancer. PI3-Ks are intracellular signal transduction molecules stimulated by various receptor tyrosine kinases (RTKs), G protein-coupled receptors, cytokine receptors as well as Ras GTPases. The activation of PI3-Ks leads to the generation of inositol lipids phosphorylated at the 3′ position of the inositol ring to generate specific phosphoinositide forms, such as 3-phosphoinositides PI(3,4)P2 and PI(3,4,5)P3 [8, 9]. PIP3 production results in the recruitment of effector proteins which harbor a pleckstrin homology (PH) domain to the plasma membrane, including serine/threonine kinases of the AGC kinase family as well as GEFs and GAPs [10]. The most prominent member of AGC serine/threonine kinases, which is linked to cellular transformation and tumorigenesis, is the Akt (PKB) kinase family comprising Akt1 (PKBα), Akt2 (PKBβ), and Akt3 (PKBγ). Activation of PI3-K results in translocation of inactive Akt to PIP3-enriched plasma membrane domains and its activation by phosphorylation. Two phosphorylation events are necessary for maximal activation of Akt, namely phosphorylation at Thr308 and Ser473 in Akt1 (Thr309/Ser474 in Akt2 and Thr305/Ser472 in Akt3) by phosphoinositide-dependent protein kinase 1 (PDK1) and the mechanistic target of rapamycin (mTOR) complex 2 (mTORC2), respectively [11, 12]. Although the three isoforms differ in their structure, all isoforms are very similar in their phosphorylation sites. Thus, phosphorylation of Akt at the serine residue serves as a surrogate marker of class I PI3-K activation, since activated Akt can phosphorylate substrates up to 2 h post-stimulation [13]. In the fully active state Akt translocates from the membrane into the cytoplasm or the nucleus and phosphorylates numerous substrates encompassing the consensus motif R-X-R-X-X-S/T-Φ [10, 14]. More than 100 substrates are known up to date, which are involved in regulating numerous cellular functions, such as proliferation, growth, metabolism, cell migration and invasion, pointing towards a key function of Akt kinases in cell physiology and pathophysiology [15]. Thus, hyperactivation of Akt and Akt-dependent signaling pathways may well promote tumor cell proliferation, survival, and tumor progression [16]. During the last 20 years of investigation more and more evidence has emerged that the three Akt isoforms have unique, non-redundant functions. By creating knockout mouse models, it was shown that Akt1 is important for cell growth and proliferation as well as neonatal development, whereas Akt2 regulates the glucose metabolism. For Akt3 a pivotal role in brain development was discovered. Moreover, distinct and partially opposing effects were found in cancer cells and tumorigenesis, thus far best analyzed in breast cancer (summarized in [15, 17]). Here, Akt2 supports migration and invasion, whereas Akt3 exhibits pro-proliferative and anti-metastatic effects. It is of note that cell-type and tumor-type specific effects for the individual Akt isoforms have been reported, but isoform-specific targets mediating tumor promoting or suppressing processes have also not been characterized [18, 19]. An influence of oncogenic K-Ras on Akt-isoforms is currently not well analyzed. In NSCLC cells with oncogenic K-Ras, inhibition of Akt1 promotes migration and invasion, whereas migration is suppressed in K-Ras wildtype-expressing NSCLC cell lines [20].

Our group and others have shown that oncogenic K-Ras promotes migration and invasion and destabilizes cell-cell adhesion in pancreatic carcinoma cells [21–23]. For PANC-1 pancreatic carcinoma cells, we discovered that stable expression of constitutive active EGFP-K-Ras(V12) decreased Rac1 and RhoA activity and increased p38 MAPK activity, which contributes to enhanced cell motility and invasiveness [21]. Moreover, oncogenic K-Ras(V12) induced the expression of NCAM and polysialyltransferases in pancreatic carcinoma cells, which leads to reduced E-cadherin adhesiveness and increased cell migration by interaction of polysialylated NCAM to E-cadherin and sterical inhibition of homophilic E-cadherin trans-interactions [22]. Besides this NCAM – E-cadherin interaction, the function of E-cadherin can be abrogated by deletion, mutation, or silencing due to promoter hypermethylation of *CDH1* [24, 25]. Loss of epithelial markers like E-cadherin or the cytokeratin-mediated cellular cytoskeleton is a key process in epithelial to mesenchymal transition (EMT), which is a hallmark in metastasizing tumors derived from epithelial tissues [26, 27]. In parallel, an upregulation of mesenchymal characteristics can be observed, which are linked to marker proteins such as N-cadherin or vimentin. In vitro studies demonstrated the co-expression of epithelial and mesenchymal markers and stepwise transition in breast, ovarian, and lung cancer cell lines [28, 29]. Although some explanations have been developed how EMT can be induced during tumor progression, tumor type specific molecular mechanisms are still elusive. One important regulatory tool comprises changes in transcription factor (TF) expression and activity. A network of such transcription factors, collectively termed as EMT-TFs, especially of the Snail, ZEB, and Twist families are directly regulating gene expression of proteins of cellular adhesion, cytoskeletal organization, and cellular migration [26, 30]. Understanding the molecular mechanisms involved in regulation of E-cadherin expression and function is critical in understanding EMT.

Aim of the study was to identify mechanisms by which oncogenic K-Ras regulates E-cadherin expression and tumor cell migration. We identified the PI3-Kα-Akt signaling pathway as a crucial mediator of K-Ras(V12) and demonstrate that the three Akt isoforms were differentially regulated by this oncogene. Moreover, Akt3 was identified as a key regulator of cell-cell adhesion by regulating E-cadherin and NCAM expression.

## Materials and methods

### Antibodies, inhibitors, activators, growth factors

All antibodies are listed in the Supplementary Table S1.

 Inhibitors/activators: Mitomycin C was purchased from Santa Cruz Biotechnology (Heidelberg, Germany), LY294002 (#270–038-M005) from Alexis Biochemicals (Grünberg, Germany), Akt activator SC-79 (#123871) from Merck Chemicals (Schwalbach, Germany).

 Growth factors: Recombinant (rec.) human epidermal growth factor (#AF-100-15) and Insulin-like growth factor-1 (IGF-1) (#100–11) were obtained from PeproTech (Hamburg, Germany).

### Plasmids and primers

Plasmids: pEGFP/K-Ras(V12), pEGFP/H-Ras(V12), pEGFP/N-Ras(V12), pVL1393/HA-K-Ras(V12), pVL1393/HA-H-Ras(V12), pVL1393/HA-N-Ras(V12) were cloned as described in Meinohl et al., 2019 [31]. Cloning of p(ΔEGFP)/TAP-EGFP-K-Ras: The TAP-tag was amplified from the plasmid pBS1761 (Cellzome, Heidelberg, Germany) [32] using the 5’primer: 5′-AGGTACCCGGGATGGCAGGCCTTGCGCAACA-3′ and 3’primer: 5′-TCGACGGGATCCATGGGCTTATCGTCAT-3′, subcloned into pCR-Blunt and then ligated as SmaI/BamHI fragment into pKS. The coding sequence of EGFP-K-Ras(V12) was isolated as NcoI/BamHI fragment from pEGFP-C3/K-Ras(V12) and the coding sequence of EGFP was isolated from pEGFP-N1 as NcoI/NotI fragment and both were ligated into pKS/TAP. The cDNA of TAP-EGFP-K-Ras(V12) and TAP-EGFP was then inserted as SmaI/NotI fragment into pEGFP-N1 in which the EGFP sequence was excised by SmaI/NotI restriction to yield p(ΔEGFP)/TAP-EGFP-K-Ras(V12) and p(ΔEGFP)/TAP-EGFP. The plasmid p(ΔEGFP)/TAP-EGFP-K-Ras(S17N) was generated by exchanging K-Ras(V12) by K-Ras(N17) as NdeI/BamHI fragment.

pLKO.1-puro_shAKT1 (SHCLND-NM_005163), pLKO.1_puro_shAKT2 (SHCLND- NM_001626), pLKO.1_puro_shAKT3 (SHCLND- NM_005465), and the non-target shRNA containing vector pLKO.1_puro_scrambled (SHC002) were purchased from Sigma Aldrich (Taufkirchen, Germany). Third-generation lentiviral packaging plasmids pCMV-VSV-G, pMDLG/PRRE, and pRSV-REV were purchased from Addgene (Watertown, USA).

### qPCR primers

AKT1: forward: 5′-GCTGACGGCCTCAAGAAGCA-3′, reverse: 5’ACCTTGCCGAAAGTGCCCTT-3′; AKT2: forward: 5′-CCGCCTGTGCTTTGTGATGG-3′, reverse: 5′-TTTCCAGCTTGATGTCGCGG-3′; AKT3: forward: 5′-ACAGATGGCTCATTCATAGG-3′, reverse: 5′-CCCTTTCCTCTGGAGTATCT-3′: RPLP0: forward: 5′- GTCGGAGGAGTCGGACGAG-3′, reverse: 5′-GCCTTTATTTCCTTGTTTGTCAAA-3′, synthesized by BiomersNet GmbH (Ulm, Germany).

QuantiTect Primer (Qiagen, Hilden, Germany): Hs_RPLP0 (QT00075012); Hs_NCAM1 (QT00071211); SNAI2 (Slug) (QT00044128); Hs_ZEB1 (QT00020972), HS_CDH1 (E-cadherin) (QT00080143).

### Cell lines and culture conditions

The human pancreatic carcinoma cell line PANC-1, the human embryonic kidney cell line HEK293, the human lung carcinoma cell line H23 (all from the American Type Culture Collection, USA) and Colo-699 (DZMS, Germany) were maintained in DMEM supplemented with 10% fetal calf serum (FCS) (Capricorn, Ebsdorfergrund, Germany), 2 mM L-glutamine and 0.1 mM MEM non-essential amino acids solution (ThermoFisher Scientific, Langenselbold, Germany) at 37 °C in a humidified atmosphere with 10% CO_2_ and routinely tested for mycoplasma contamination. Serum-starvation was performed overnight in DMEM without supplements. PANC-1 cell clones stably expressing EGFP, EGFP-K-Ras(V12), EGFP-K-Ras(N17), EGFP-N-Ras(V12), or EGFP-H-Ras(G12) were generated and cultured as described in Schreiber et al., 2008 [22]. Transfected HEK293 and Colo699 cell clones were generated according to protocols herein. To generate Akt isoform knockdown (Akt-kd) cell clones, H23, PANC1/EGFP and PANC-1/EGFP-K-Ras(V12), were transduced with an AKT isoform shRNA containing vector [33] and selected by addition of 1.5 μg/ml puromycin (Sigma-Aldrich, Taufkirchen, Germany). Recombinant baculoviruses expressing HA-tagged Ras variants were generated by transfecting *Spodoptera frugiperda* 9 (*Sf*9) (ThermoFisher Scientific, Langenselbold, Germany) insect cells with pVL1393 encoding HA-H-Ras(V12), HA-K-Ras(V12), and HA-N-Ras(V12) as described in Meinohl et al., 2019 [31]. All other cell lines used are listed in Table S2 and cultured in DMEM supplemented with 10% FCS, 2 mM L-glutamine and 0.1 mM MEM non-essential amino acids at 37 °C, 10% CO_2._

### siRNA transfection

For siRNA transfection of EGFP- and EGFP-K-Ras(V12)-expressing PANC-1 cells 1.5 × 10^5^ cells/chamber were seed in Lab Tek chambered coverglass slides (2-chamber) (Nunc, Kamstrup, Denmark) and cultured for 24 h. For transfection 3.75 μl of DMRIE-C Reagent, 75 μl OptiMEM-I (Invitrogen, Groningen, Netherlands), 66 nM siAkt or fluorescein-conjugated control siRNA (SignalSilencer Akt siRNA I, #6211, SignalSilencer Control siRNA #6201; Cell Signaling Technology, Leiden, Netherlands), and 375 μl growth medium was used. The cells were incubated for at least 72–96 h to obtain sufficient inhibition of protein expression. Wounding assays were started 48 h after transfection.

### Stable Akt isoform specific knockdown

HEK293T cells were transfected with shRNA containing vectors purchased from Sigma-Aldrich (Taufkirchen, Germany) targeting either AKT isoforms (pLKO.1-shAKT1-puromycin, pLKO.1-shAKT2-puromycin, pLKO.1-shAKT3-puromycin) or scrambled control (pLKO.1-non-target-puromycin). Packaging plasmids for third generation lentiviral transfection (pCMV-VSV-G, pMDLG/PRRE, pRSV-REV (Addgene, Watertown, USA) were used. The transfection rate was enhanced using the Lipofectamine 3000 transfection reagent according to the manufacturer’s instructions (ThermoFisher Scientific, Langenselbold, Germany). Every 24 h the lentiviral supernatants from the HEK293T cells were collected, filtered through a 0.22 μm filter and 2 ml of the supernatant was added to the target cell lines. This step was subsequently repeated three times using the 24 h, 48 h, and 72 h supernatant past transfection. Transduction efficacy was enhanced using 8 μg of hexadimethrine bromide (Sigma Aldrich, Taufkirchen, Germany). On the fourth day of transduction the cells were selected by adding 1.5 μg/ml puromycin to the respective standard cell culture media for at least 2 weeks. Successful knockdown of the specific Akt isoforms was confirmed by Western blot analysis using isoform specific antibodies.

### Identification of Ras interacting proteins

HA-K-Ras(V12), HA-H-Ras(V12), and HA-N-Ras(V12) were expressed in baculovirus-infected *Sf*9 insect cells and purified from the particulate (P100) membrane-containing fraction with anti-HA (12CA5) antibody (Roche, Mannheim, Germany) covalently linked to Protein A Sepharose® Cl-4B (GE Healthcare, Braunschweig, Germany). Purified HA-tagged Ras proteins (500 ng) were immunoprecipitated with μMACS HA Isolation Kit as described in Meinohl et al., 2019 [31]. Precleared PANC-1 cell lysate (10 mg) was added to immobilized HA-Ras proteins and incubated for 2 h under constant rotation. Protein complexes were purified on μColumns (Miltenyi Biotec, Germany) as described in Meinohl et al., 2019 [31]. The eluted proteins were separated on 10% SDS-polyacrylamide gels and either silver-stained or subjected to Western blotting using anti PI3-K p85α antibody and ECL detection.

### Tandem affinity purification system (TAP system)

TAP fusion proteins were transiently expressed in PANC-1 cells for 24 hours. Therefore, 3 × 10^6^ PANC-1 cells were transfected with 8 μg of plasmid DNA and 30 μl of DMRIE-C reagent (ThermoFisher Scientific, Langenselbold, Germany) in serum-free DMEM. The medium was replaced by FCS-containing medium after 6 h and cells were lysed 24 h after transfection in Gold-Lysis buffer [20 mM Tris/HCl, pH 7.9; 50 mM NaCl; 10% (V/V) glycerin; 1% (V/V) TritonX-100; 1 mM PMSF; 1 mM Aprotinin; 10 μM Leupeptin; 1 μM Pepstatin A; 1 mM EGTA; 1 mM sodium orthovanadate; 10 mM sodium fluoride; 0.5 μM GTP]. Dependent on the expression of the TAP fusion protein, 1–5 mg of cell lysate was incubated with 100 μl of IgG Sepharose beads (GE Healthcare, Braunschweig, Germany) in 1.8 ml Gold-Lysis buffer for 4 h at 4 °C by end-over-end rotation. After washing the IgG beads three times with 2 ml of Gold-Lysis buffer with 150 mM NaCl and two times with TEV buffer [50 mM Tris/HCl, pH 8,0] they were resuspended in 130 μl of this. Subsequently, 20 μl of TEV protease (10 U/μl, Invitrogen, Groningen, The Netherlands) were added and incubated for 2 h at 30 °C with constant rotation. Finally, the reaction mixture was analyzed by SDS-polyacrylamide gels and Western blotting.

### Protein lysate preparations

Cells were lysed by scraping them into RIPA buffer or NP-40 buffer. *RIPA buffer*: 50 mM Tris/HCl, pH 7.2; 150 mM NaCl; 0.1% (V/V) SDS; 0.5% (m/V) sodium deoxycholate; 1.39 mM Pefabloc; 15 μM Aprotinin; 21 μM Leupeptin; 50 μM trypsin inhibitor; 10 mM sodium pyrophosphate; 25 mM β-glycerophosphate; 2 mM sodium orthovanadate; 10 mM sodium fluoride; *NP-40 buffer:* 50 mM HEPES, pH 7.5; 150 mM NaCl; 1% (V/V) NP-40; 2 mM EDTA; 10% (V/V) glycerin; 1 mM PMSF; 2.1 μg/ml Aprotinin; 1 mM Leupeptin; 50 mM sodium fluoride; 10 mM sodium pyrophosphate; 1 mM sodium orthovanadate. Cells were homogenized by forcing the suspension 5–10 times through a 0.45 × 25 mm needle attached to a syringe and finally cleared by centrifugation at 16,100 x g for 15 min at 4 °C. 30–50 μg of protein lysate were analyzed by Western blotting, unless otherwise stated. Phosphorylated and non-phosphorylated proteins were detected using specific antibodies and either ECL Western Blotting Detection System (GE Healthcare, Braunschweig, Germany), Fusion Imaging System and Software (Vilbert Lourmat, Eberhardzell, Germany), or Odyssey Infrared Imaging System and Software (LI-COR Biosciences, Bad Homburg vor der Höhe, Germany).

### In vitro Akt kinase activity assays

The kinase activity of the three different Akt isoforms was analyzed in vitro according to Grabinski et al., 2011 [34] with some modifications. Briefly, each Akt isoform was immunoprecipitated from 250 to 1500 μg of cell lysate, prepared in NP-40 buffer, with 1.5 μg of an isoform specific antibody: anti-Akt1 (2H10) (#2967), anti-Akt2 (L79B2) (#5239) (Cell Signaling Tech., Leiden, The Netherlands), anti-Akt3 (GMA104) (#05–780; Merck Millipore, Darmstadt, Germany). Mouse IgG2a (#X0943; DAKO, Hamburg, Germany) served as a negative control. The antibodies were coupled to 25 μl of Protein G-Agarose beads for 2 hours at 4 °C under constant rotation prior to addition of the lysate. Immunoprecipitation of Akt was carried out for 2 h at 4 °C and constant end-over-end rotation. For the kinase activity assay, the beads were washed twice with 500 μl NP-40 buffer and once with *Kinase buffer:* 25 mM Tris/HCl, pH 7.5; 10 mM MgCl_2_; 5 mM β-glycerophosphate; 0.1 mM sodium orthovanadate, 2 mM DTT. The precipitate was dried with a Hamilton syringe and 20 μl of *Kinase Reaction Mix* [1 x Kinase buffer, 0.2 mM ATP, 1 μg GSK-3α/β-GST-fusion protein (Cell Signaling Tech., Leiden, The Netherlands)] or GSK-3α fusion protein (#PK-CA577–7003, PromoKine, Heidelberg, Germany)] was added. The reaction mix was incubated for 30 minutes at 30 °C. Finally, 5 μl of 10x SDS loading buffer were added, the solution was heated 5 minutes at 95 °C and analyzed by SDS-PAGE and immunoblotting procedure. Phosphorylation of GSK-3α as Akt substrate was detected by phospho-GSK-3α/β (Ser21/9) antibody (#9331, Cell Signaling Tech., Leiden, The Netherlands), peroxidase-conjugated secondary antibody, and ECL detection.

### Subcellular fractionation

To differentiate membrane associated, particulate (P100) and soluble proteins (S100), subcellular fractions were prepared by centrifugation of cell lysates at 100,000 x g in HEPES buffer as described in Dreissigacker et al., 2006 [21]. [*HEPES buffer*: 50 mM HEPES, pH 7.6; 8.6% (m/v) sucrose; 10 mM EDTA; 10 mM EGTA; 1.39 mM Pefabloc; 15 μM Aprotinin; 21 μM Leupeptin; 50 μM trypsin inhibitor]. To distinguish between actin-associated and soluble proteins, Triton X-100 fractionation was performed. Cells were incubated for 15 min on a rocking platform in *Triton lysis buffer:* 25 mM HEPES, pH 7.4; 1% (v/v) Triton X-100; 0.3 M sucrose; 104 mM NaCl; 5 mM KCl; 1 mM KH_2_PO_4_; 1.2 mM MgCl_2_; 2 mM CaCl_2_; 1.39 mM Pefabloc; 15 μM Aprotinin; 21 μM Leupeptin; 50 μM trypsin inhibitor. The suspension was centrifuged at 13,000 x g for 10 min at 4 °C and the supernatant represents the Triton-soluble fraction. The pellet containing Triton-insoluble, actin cytoskeleton-associated proteins, was resuspended in RIPA buffer. All fractions were analyzed by SDS-PAGE and immunoblot procedure.

### Wounding assays

For wound healing assays, cells were seeded into Lab Tek chambered coverglass slides (2-chamber, 1.2–2 × 10^5^ cells/chamber; Nunc, Kamstrup, Denmark) or into six-well plates (8 × 10^5^ cells/well) and cultured for 24–48 h until confluent. Cells were treated with mitomycin-C (10 μg/ml) for 2 h to inhibit proliferation. Parallel scratches (250–300 μm) were made into the confluent monolayer using a pipette tip. The cells were washed two times with medium to remove detached cells and then incubated in DMEM without supplements and 10 μM LY294002 or methanol as solvent, when indicated. Transfection with 66 nM Akt siRNA was performed 48 h prior to wounding. The wound closure of cells grown in chamber slides was documented by taking images directly (t_0_), at 12 h (t_12_), 24 h (t_24_), and 48 h (t_48_) after wounding with an Olympus IX70 microscope (Olympus, Hamburg, Germany) at three randomly chosen areas using a CCD camera and analySIS 3.2 imaging system (Soft Imaging System, Münster, Germany). The gap size was measured using analySIS software and the wound closure calculated in percent. Wounding assays performed in six-well plates were evaluated using EVOS FL Auto 2 Cell Imaging System (ThermoFisher Scientific, Langenselbold, Germany), analyzed by Scratch Assay Analyzer Plugin (MiToBo, Universität Halle, 10.1016/j.patcog.2012.03.001), and cell migration was calculated in μm/h as described in Meinohl et al., 2019, [31].

### Quantitative RT-PCR studies

Total RNA was prepared from confluent cell layers using the RNeasy Mini Kit (Qiagen, Hilden, Germany) according to the manufacturer’s instructions. Synthesis of cDNA from 1 μg of RNA was performed with oligo-dT primers and SuperScript™ II Reverse Transkriptase (Invitrogen, Karlsruhe, Germany). SYBR green-based, qRT-PCRs were performed using QuantiTect primer pairs (Qiagen) or short primers synthesized by Biomers.net (Ulm, Germany). Each sample was analyzed in duplicate as described in Seiz et al., 2020, [35]. Detection of RPLP0 was used as a reference. The dissociation curves were calculated using the MxPro qPCR software (Agilent Technologies, Waldbronn, Germany) and the amplification was calculated by the 2-ΔΔCt method.

### Miscellaneous

Protein concentration was determined by the bicinchoninic acid (BCA) assay (Pierce, Sankt Augustin, Germany). For immunoblotting equal loading and transfer of proteins was controlled by Ponceau S staining (Sigma-Aldrich, Taufkirchen, Germany). All experiments were repeated as indicated and identical trends or similar results were obtained each time. Data from representative experiments are shown. The quantification of Western blots was performed using Image Studio Lite Software (Li-COR Biociences, Bad Homburg vor der Höhe, Germany).

All statistical analyses were performed with GraphPad Prism 9.5.1 software (GraphPad Software Inc., San Diego, CA, USA). All depicted values represent the means ± SD of at least 3 independent experiments. For statistical analysis Gaussian distribution of the data was tested (Shapiro–Wilk test or Kolmogorov-Smirnov test). The statistical significance was determined by using appropriate tests: the One sample t and Wilcoxon test, the unpaired t-test (two-tailed) or the One-way ANOVA for multiple comparison. A value of *p* ≤ 0.05 (*) was considered as significant.

## Results

### PI3-kinase α is a K-Ras(V12) effector and mediates Akt-phosphorylation

**Fig. 1:**
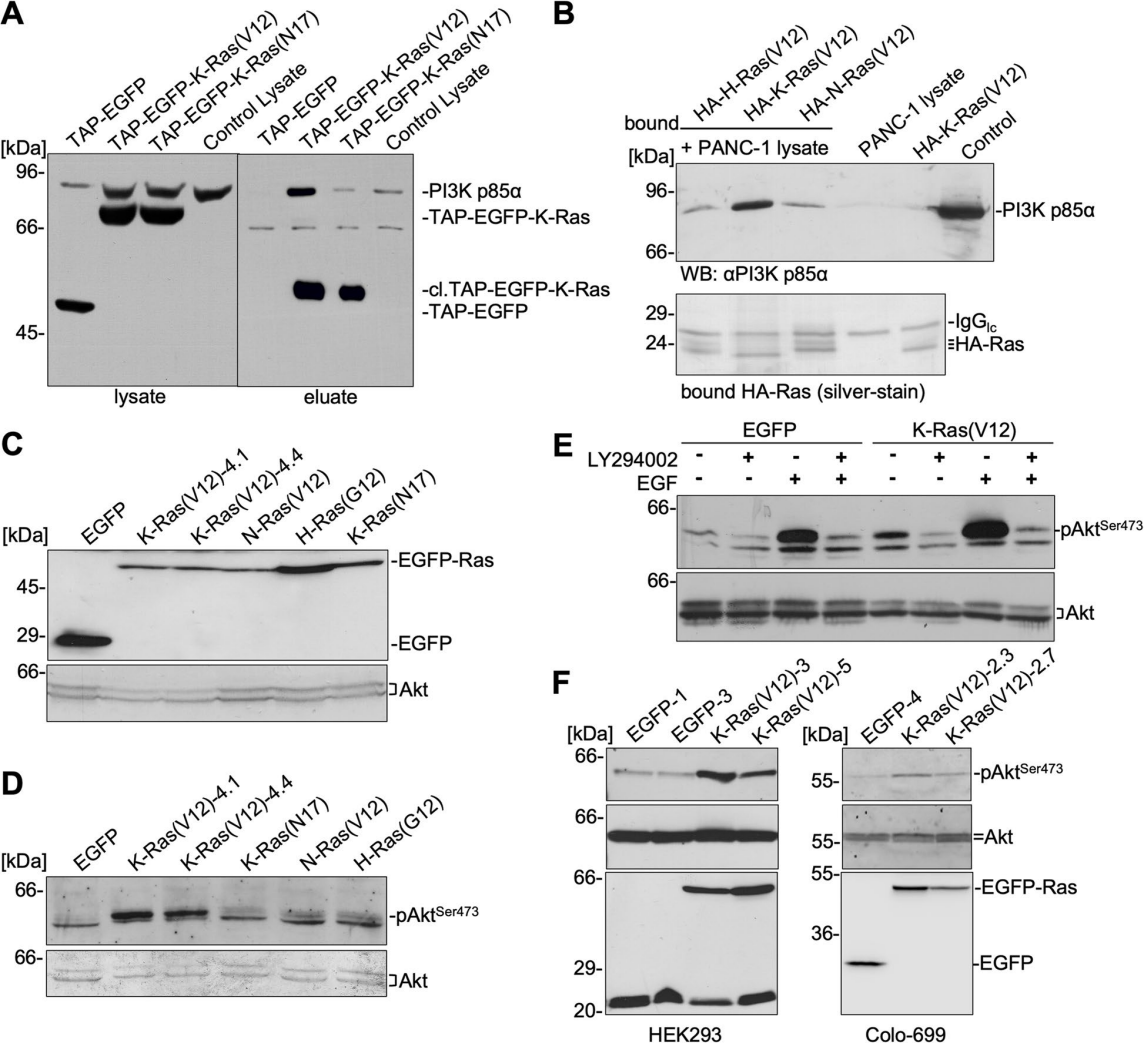
K-Ras(V12) interacts with PI3-Kα and activates Akt. **A**, **B** Interaction of K-Ras(V12) with PI3-K. **A** PANC-1 cells, transiently expressing TAP-EGFP, TAP-EGFP-K-Ras(V12), or TAP-EGFP-K-Ras(N17), and non-transfected control cells were lysed 24 h after transfection. TAP-containing proteins were precipitated from 5 mg of cell lysate (1 mg for TAP-EGFP) by binding to IgG sepharose beads and eluted by cleavage of the TAP tag with TEV protease. Lysates and eluates were analyzed by SDS PAGE and Western blotting using anti-p85α PI3-K or anti-GFP antibody and ECL. The left panel [lysate] shows the expression of the TAP-EGFP proteins and endogenous PI3-K p85α subunit in 8 μg (first lane) or 40 μg of lysate. The right panel [eluate] shows the precipitated, TEV-cleaved (cl.) TAP-proteins and demonstrates that PI3-K co-precipitated only with TAP-EGFP-K-Ras(V12). One representative assay (*n* ≥ 3) is shown. **B** Purified, post-translationally modified HA-Ras proteins [HA-H-Ras(V12), HA-K-Ras(V12), HA-N-Ras(V12), 500 ng each], were immobilized on anti-HA μMacs beads and incubated with PANC-1 cell lysate [lanes 1–3]. As controls, anti-HA μMacs beads incubated with PANC-1 lysate [PANC-1 lysate] or purified HA-K-Ras(V12) [HA-K-Ras(V12)], and to determine endogenous PI3-K p85α an aliquot of PANC-1 lysate (50 µg)  [Control] were used. The Western blot in the upper panel demonstrates the detection of the co-precipitated PI3-K whereas the lower panel shows a silver-stained gel with one third of the precipitated HA-Ras and IgG (light chain) proteins (*n* = 3). **C**-**F** Activation of Akt by EGFP-K-Ras(V12). **C** PANC-1 cells and PANC-1 cell clones stably expressing EGFP, EGFP-K-Ras(V12) (clone 4.1 and 4.4), EGFP-N-Ras(V12), EGFP-H-Ras(G12), or EGFP-K-Ras(N17) were analyzed for expression of EGFP, EGFP-Ras proteins, and Akt by Western blotting. **D** The amount of pAkt^Ser473^ (upper blot) and total Akt (lower blot) was detected by Western blotting in serum starved EGFP and EGFP-Ras cell clones. pAkt^Ser473^ is increased in K-Ras(V12)-expressing PANC-1 cells (*n* > 3). **E** Serum-starved PANC-1 cell clones were incubated with 10 μM LY294002 for 20 min, 30 ng/ml EGF for 10 min, or with EGF after preincubation with LY294002. Cell lysates were subjected to Western blotting for detection of pAkt^Ser473^ and Akt (*n* > 3). Inhibition of PI3-K reduced basal as well as EGF-induced Akt phosphorylation. **F** Lysates of HEK293 and Colo-699 cells stably expressing EGFP or EGFP-K-Ras(V12) were subjected to Western blotting. The amount of pAkt^Ser473^, Akt, EGFP-Ras, and Ras was detected by using appropriate antibodies demonstrating that pAkt is increased in K-Ras(V12)-expressing cells

The tandem affinity purification system (TAP system) represents an ideal method to analyze protein interactions and protein complexes that are not particularly abundant under native conditions [36, 37]. To detect K-Ras(V12) binding proteins, TAP-tagged constitutive active K-Ras(V12) and dominant negative K-Ras(N17) were transiently expressed in PANC-1 cells and precipitated by binding to an IgG affinity matrix. TAP-K-Ras protein complexes were eluted by cleavage of the TAP-tag with TEV protease. The eluates comprising the calmodulin binding peptide (CBP) fusion proteins and their interacting proteins were analyzed by Western blotting. To control for the binding of TAP-tagged complexes to the IgG beads, the precipitates were additionally analyzed without eluting the proteins by TEV protease cleavage (data not shown). Figure 1A shows that PI3-kinase α (PI3-Kα) was detected specifically in the eluate of TAP-EGFP-K-Ras(V12) using an antibody against the p85α subunit but hardly in the corresponding eluate of TAP-EGFP-K-Ras(N17), TAP-EGFP expressing cells or non-transfected PANC-1 cell lysates. Quantification of the amount of PI3-K/p85α by densitometric analysis of four independent precipitation assays revealed a binding of p85α to TAP-EGFP-K-Ras(N17) of 12.04% ± 3.76% related to the binding to TAP-EGFP-K-Ras(V12) set 100%. When using lysates of non-transfected PANC-1 cells (Control lysate), an unspecific binding of p85α to the IgG Sepharose beads of 17.81% ± 8.94% was evident compared to TAP-EGFP-K-Ras(V12) set 100%. These results demonstrate that PI3-Kα is a bona fide effector of active K-Ras4B since it interacts only with active, GTP-bound K-Ras(V12). The activity of K-Ras(V12) and K-Ras(N17) was verified by Ras activity assays using the Ras-binding domain (RBD) of Raf-1 as an activation-specific probe for Ras-GTP (data not shown). We have recently identified K-Ras4B binding proteins in co-immunoprecipitation (co-IP) experiments by using recombinant, post-translationally modified HA-tagged Ras proteins expressed in baculovirus-infected *Sf*9 insect cells [31]. To determine whether PI3-Kα also interacts with the other Ras isoforms, purified HA-tagged H-Ras(V12), K-Ras(V12), and N-Ras(V12) were immobilized on anti-HA magnetic μMacs beads. These complexes were then used as bait to precipitate PI3-Kα from PANC-1 cell lysates (Fig. 1B). In the upper panel it is shown that the p85α subunit of PI3-Kα was markedly precipitated by HA-K-Ras(V12) but considerably less by HA-H-Ras(V12) and HA-N-Ras(V12). The lower panel shows the amount of the precipitated HA-Ras isoforms and confirmed equal amounts of bait proteins.

Next, we analyzed whether the interaction of K-Ras(V12) and PI3-Kα resulted in an activation of the PI3-K-Akt pathway. The expression of Akt and the phosphorylation at Ser473 was detected by Western blotting in PANC-1 cell clones stably expressing EGFP, EGFP-K-Ras(V12) (clone 4.1 and 4.4), EGFP-N-Ras(V12), EGFP-H-Ras(G12), and EGFP-K-Ras(N17). Figure 1C shows the expression of EGFP proteins and of Akt. Figure 1D illustrates a distinctly upregulated Akt phosphorylation at Ser473 in the two cell clones expressing K-Ras(V12) and to a lower extent in N-Ras(V12) clone. Densitometric analyses of pAkt/Akt intensities of 3–4 independent assays revealed a significant increase for K-Ras(V12) as compared to the EGFP control; specifically: 4.59 ± 0.69, (*n* = 3, *p* = 0.012, one sample t-test) for clone 4.1 and 4.81 ± 2.29 (*n* = 4, *p* = 0.045) for clone 4.4. For N-Ras(V12) a 2.04 ± 1.27, for H-Ras a 1.73 ± 1.08 and for K-Ras(N17) a 1.31 ± 0.83-fold change (for all: *n* = 4, ns) was determined. Note, that the K-Ras(V12)-expressing cells express less Akt (Fig. 1C) as well as PI3-K p85α (Fig. S1A) than the EGFP and parental PANC-1 cells. Akt expression was densitometrically quantified and the comparison to the EGFP control revealed for both K-Ras(V12) clones a significant 50% reduction with a fold change of 0.56 ± 0.14 (*n* = 4, *p* = 0.0085, one sample t-test) for clone 4.1 and 0.51 ± 0.11 (± SD, *n* = 5, *p* = 0.0006, one sample t-test) for clone 4.4. Akt expression was not changed in the EGFP-N-Ras(V12), -H-Ras and -K-Ras(N17) expressing clones compared to the EGFP control cells.

To confirm that the enhanced phosphorylation is mediated by PI3-K, EGFP-K-Ras(V12)- and EGFP-expressing cells were incubated with 10 μM LY294002 to inhibit basal and EGF-induced PI3-K activity. Figure 1E demonstrates, that the basal K-Ras(V12)- as well as the EGF-induced Akt phosphorylation at Ser473 was inhibited by LY294002. Similar results were obtained when the phosphorylation of Akt at Thr308 was analyzed (data not shown). Moreover, the phosphorylation of S6 ribosomal protein (S6) was also increased in K-Ras(V12)-expressing PANC-1 cells and sensitive to PI3-K inhibition (data not shown).

Enhanced phosphorylation of Akt after stable expression of EGFP-K-Ras(V12) was not only evident in PANC-1 pancreatic carcinoma cells, but also in HEK293 and Colo-699 cells (Fig. 1F). In HA-K-Ras(V12) expressing HEK293 cells pAkt was increased by 4.30 ± 1.27-fold (*n* = 3; *p* = 0.046, one sample t-test) in clone 3 and by 3.01 ± 0.50-fold (*n* = 3; *p* = 0.02) in clone 5. In Colo-699 K-Ras(V12) cell clone 2.3 pAkt was increased by 5.07 ± 1.96-fold (*n* = 3; *p* = 0.03) and in clone 2.7 by 3.99 ± 1.76-fold (*n* = 3; *p* = 0.04). These results suggest, that PI3-Kα is a K-Ras(V12) effector and mediates Akt-activation and downstream signaling.

### K-Ras(V12)-induced PI3-K-Akt activation affects cell migration

**Fig. 2:**
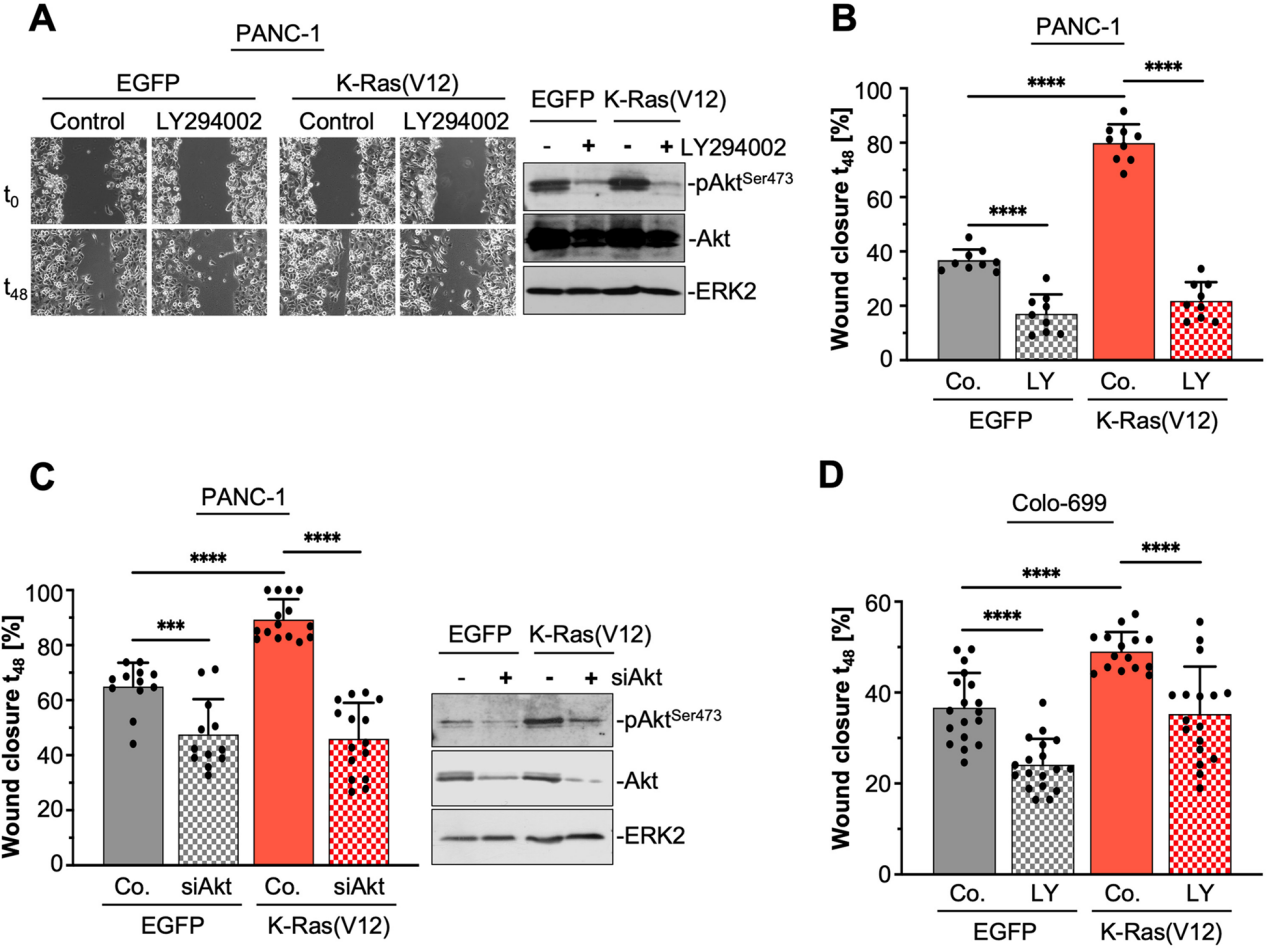
PI3-K/Akt activity modulates cell migration. **A**, **B** EGFP- and EGFP-K-Ras(V12)-expressing PANC-1 cells were incubated until confluency and proliferation was inhibited by mitomycin C. A wound of 250–300 μm was scratched into the monolayer (time point t_0_) and cells were incubated in serum-free medium with 10 μM LY294002 [LY] or solvent [Control, Co.]. Cell migration was documented after 24 h and 48 h at three randomly chosen positions. Images in **A** show representative phase contrast pictures at t_0_ and t_48_. The Western blot on the right shows the detection of pAkt^Ser473^ in lysates 48 h after wounding. Detection of ERK2 served as loading control. **B**-**D** Quantification and statistics of independent wounding assays. The size of the wound was measured and calculated as percent wound closure at t_48_. The bar graph shows the mean ± SD, the dots represent individual data points (triplicate values (B + C) or 3–5 values (D), t-test (unpaired, two-tailed), *** *p* ≤ 0.001; **** *p* ≤ 0.0001). **B** Bar graph of the quantification of the wound closure of 3 independent experiments (triplicate values) as described above. **C** Bar graph of the quantification of 4–5 independent (triplicate values) in which cells were transfected with 66 nM siAkt targeting Akt1 and Akt2. Control cells [Co.] were treated with 66 nM scrambled siRNA (siControl). Wounding was started 48 h after transfection. Quantification was performed as described above. The Western blot on the right shows the detection of pAkt^Ser473^, total Akt, and ERK2 as loading control in lysates 48 h after wounding. **D** Bar graph presenting the quantification of wounding assays with EGFP- and EGFP-K-Ras(V12)-expressing Colo-699 cell clones performed in DMEM with 5% FCS for 48 h. Quantification was conducted as described above

In previous studies [21, 22], we used trans-well cell migration assays and determined an approximately 3–4-fold increase in directed cell migration of the K-Ras(V12) expressing PANC-1 cells. To elucidate whether the K-Ras(V12)-induced PI3-K/Akt activation influences cell migration, wound healing assays were performed in the absence or presence of 10 μM LY294002. Proliferation was inhibited by mitomycin C treatment. Figure 2A shows representative phase contrast images at the time points t_0_ and t_48_, showing that the EGFP-K-Ras(V12)-expressing control cells almost completely closed the wound and were thus faster than the EGFP-expressing control cells. Migration was markedly inhibited in both cell clones by LY294002 treatment. The effectiveness of LY294002 was controlled by analyzing the inhibition of Akt phosphorylation in the cell lysates at the end of the migration experiments, as shown in the representative Western blot in Fig. 2A. The bar graph in Fig. 2B illustrates the quantification of the wound closure after 48 h. For EGFP-expressing PANC-1 cells a wound closure of 36.48 ± 4.05% without and 17.04 ± 7.13% with LY294002 treatment was determined. Thus, inhibition of PI3-K/Akt results in a ~ 53% reduction of cell migration. K-Ras(V12) expressing cells exhibit a wound closure of 79.86 ± 6.96% in the absence and 21.81 ± 6.92% in the presence of LY294002, given a ~ 73% reduction by LY294002. These results on the one hand display that the K-Ras(V12)-induced migration is more sensitive to PI3-K/Akt inhibition than the migration of PANC-1 cells, and on the other hand confirm that K-Ras(V12) expression markedly enhanced the migration, as already published in [21, 22]. Moreover, K-Ras(V12) overexpressing cells exhibit decreased proliferation [21]. Interestingly, inhibition of PΙ3-Κ had a similar effect on cell proliferation in both EGFP- and EGFP-K-Ras(V12)-expressing cells, increasing the doubling time by approximately 30% from 30 h to 41 h (EGFP) and from 35 h to 44 h (EGFP-K-Ras(V12)), respectively.

As demonstrated in Fig. 1, binding of K-Ras(V12) to PI3-K resulted in enhanced activation of Akt. To analyze the impact of Akt on migration, expression of Akt was transiently inhibited via siRNA targeting both Akt1 and Akt2. Wounding was started 48 h after transfection and continued for 48 h, with a maximum of Akt1/2 depletion between 72 and 96 h. The depletion of Akt1/2 was examined at the end of the experiment by Western blot analysis (Fig. 2C). As depicted in the bar graph, depletion of Akt1/2 reduced the migration of both EGFP-K-Ras(V12)- and EGFP-expressing cells to the same baseline. However, the inhibition was more pronounced in the K-Ras(V12)-expressing PANC-1 cells as compared to the control cells. Precisely, after 48 h EGFP-K-Ras(V12) cells exhibit 89.3 ± 7.37% wound closure with siControl, and 45.99 ± 13.06% with siAkt, thus a 43.31% inhibition. For EGFP-control cells a 17.38% inhibition of wound closure was evident (siControl: 64.92 ± 8.67%, siAkt: 47.54 ± 12.84%).

K-Ras(V12)-induced migration was also evident in stably expressing EGFP-K-Ras(V12) Colo-699 carcinoma as well as HEK293 cells. Figure 2D shows for EGFP-expressing Colo-699 cells a wound closure of 36.69 ± 7.66% without LY294002 and 24.14 ± 5.68% with LY294002 and for EGFP-K-Ras(V12)-expressing Colo-699 cells 49.05 ± 4.26% without and 35.28 ± 10.44% with LY294002. EGFP-expressing HEK293 cells exhibit a wound closure of 54.97 ± 2.20%, and for EGFP-K-Ras(V12) 68.03 ± 7.54% within 24 h (*n* = 3; *p* < 0.05; unpaired t-test).

Taken together, these results demonstrate that K-Ras(V12)-induced PI3-K/Akt activation enhances migration in all analyzed cell lines.

### K-Ras(V12) alters Akt isoform expression

**Fig. 3:**
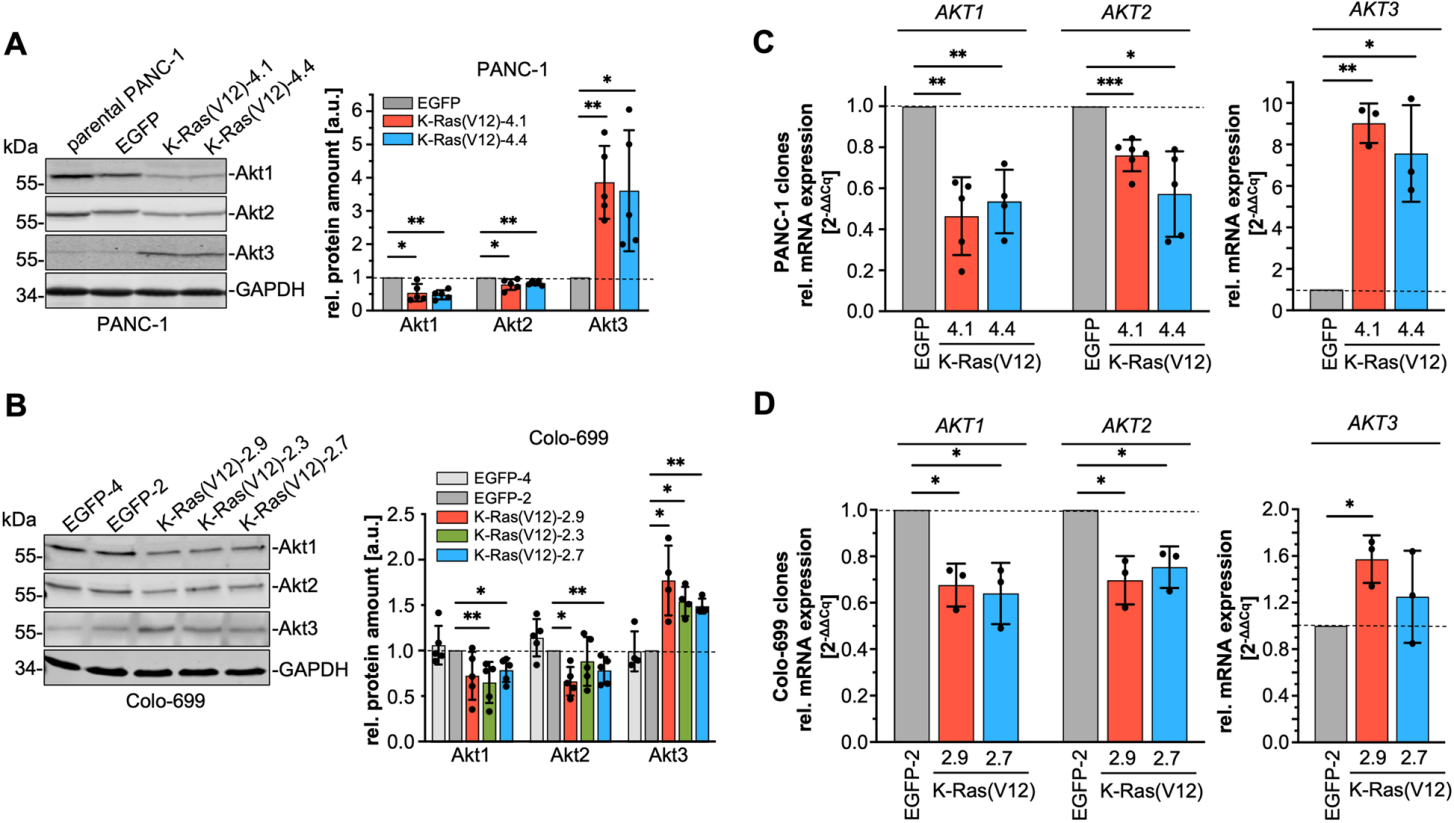
K-Ras(V12) differentially regulates Akt isoform expression. The amount of Akt isoforms was analyzed on protein (**A**, **B**) and mRNA (**C**, **D**) level in PANC-1 and Colo-699 cancer cells. **A**, **B** Akt isoforms were detected in RIPA cell lysates by Western blotting using Akt isoform-specific antibodies and GAPDH as loading control. Depicted are representative blots of at least three independent experiments. The bar graphs show the densitometric analysis as a ratio of Akt in relation to loading control normalized to an EGFP control clone 2 (mean ± SD, *n* = 4–5). **C**, **D** The mRNA content of each *AKT* variant was determined by qRT-PCR analysis. Relative expression of each *AKT* variant was assessed in relation to the expression of the house keeping gene *RPLP0* and normalized to the corresponding EGFP cell clone*.* Each sample was analyzed in duplicate using three (Colo-699) or 3–6 (PANC-1 clones) different cDNAs (mean ± SD). Statistical analyses: one sample t-test, **** *p* ≤ 0.0001; *** *p* ≤ 0.001; ** *p* ≤ 0.01; * *p* ≤ 0.05

EGFP-K-Ras(V12)-expressing PANC-1 cells express less Akt protein as compared to the control cells (Fig. 1, Fig. S1A). Western blot analyses with Akt isoform-specific antibodies revealed that the amount of Akt1 and Akt2 was decreased in both EGFP-K-Ras(V12) clones, whereas the amount of Akt3 was increased (Fig. 3A). This down- versus upregulation of Akt isoforms, especially the enhanced expression of Akt3 was not only evident in the PANC-1 cell clones, but also in three Colo-699 EGFP-K-Ras(V12)-expressing cell clones (Fig. 3B). EGFP-K-Ras(V12)-expressing HEK293 cells also exhibited less Akt1 and Akt2 protein. Akt3 was detected in a double band, which might indicate an enhanced phosphorylation of this isoform (Fig. S1B).

In the next step, quantitative reverse transcriptase-polymerase chain reaction (qRT-PCR) analyses were performed, to detect possible differences in the mRNA-expression of *AKT1, AKT2,* and *AKT3* mediating the alterations in the protein amount*.* Figure 3C demonstrates that stable expression of EGFP-K-Ras(V12) in PANC-1 cell clones reduced the mRNA of *AKT1* by ≈ 50% in both clones (0.46 ± 0.19 and 0.54 ± 0.16) and of *AKT2* by ≈ 25 and 45% (0.76 ± 0.08 and 0.57 ± 0.21). In contrast, *AKT3* mRNA was markedly increased by more than 8-fold in EGFP-K-Ras(V12)-expressing cell clones (9.02 ± 0.95 and 7.57 ± 2.33) and this increase is reflected in the enhanced protein amount (Fig. 3A). For the EGFP-K-Ras(V12)-expressing Colo-699 cells, both clones showed a reduction of *AKT1* and *AKT2* by about 35 and 30%, respectively (Fig. 3D). *AKT3* expression was enhanced in K-Ras(V12) clone 2.9 by 1.6-fold and by 1.3-fold in clone 2.7. Taken together these results demonstrate that K-Ras(V12) interacts with PI3-K, activates Akt, and differentially alters Akt isoform expression.

### Akt isoform expression and activity

**Fig. 4:**
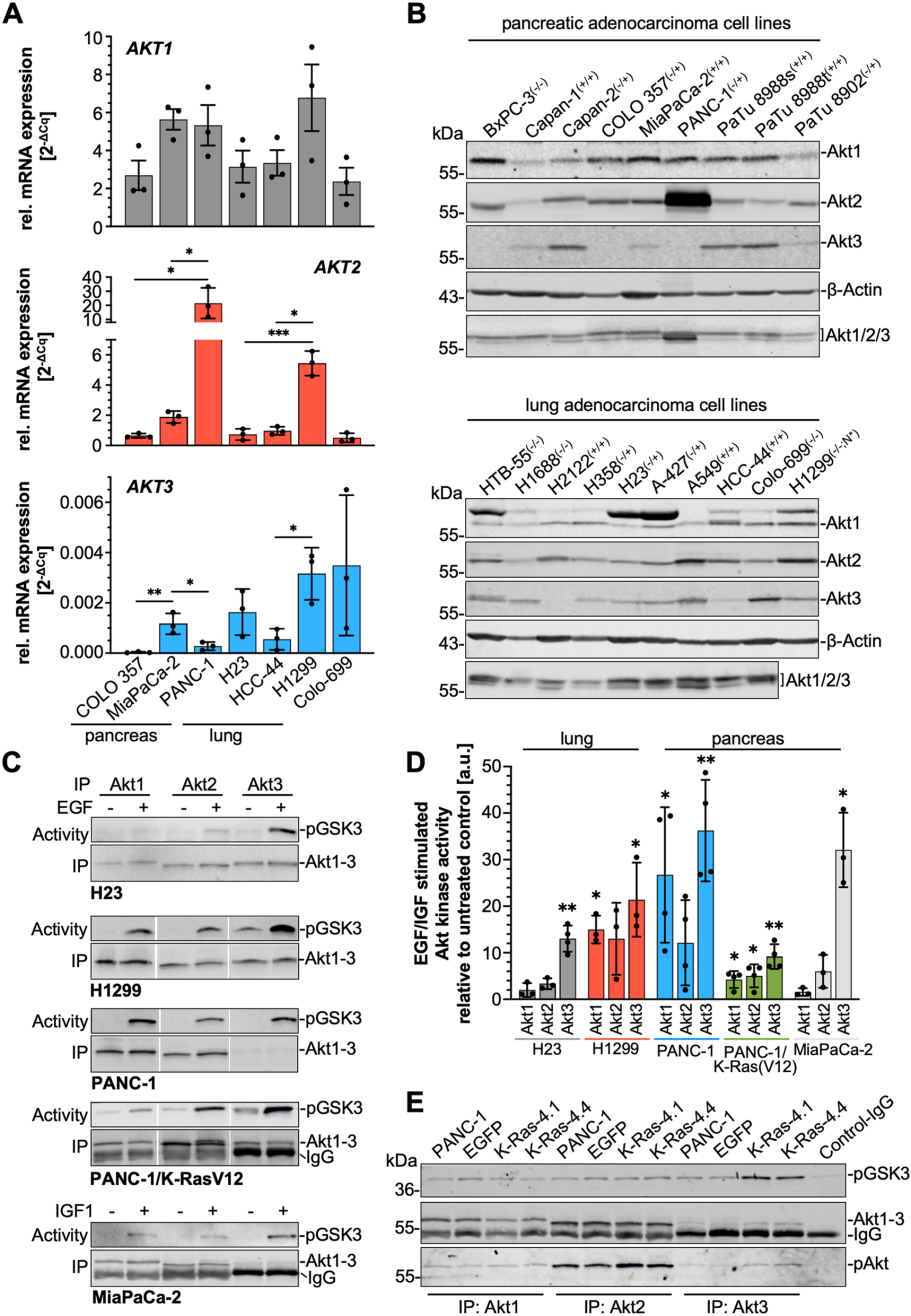
Expression and activity of Akt isoforms. **A** The amount of the mRNA of *AKT* variants in three pancreatic and three lung adenocarcinoma cell lines and Colo-699 cells was analyzed by qPCR. Relative expression of each *AKT* variant was assessed in relation to the house keeping gene *RPLP0.* Three different cDNAs were used and analyzed in duplicate, mean ± SD is shown. Statistical analyses for pancreas and lung cell lines were done separately: One-way ANOVA (with Tukey’s multiple comparison tests), *** *p* ≤ 0.001, ** *p* ≤ 0.01; * *p* ≤ 0.05. **B** The expression of Akt isoforms in nine pancreatic, nine lung adenocarcinoma cell lines and Colo-699 cells was detected in RIPA cell lysates by Western blotting using Akt and Akt isoform-specific antibodies, β-actin served as loading control. Representative blots of n ≥ 3 independent experiments are shown. K-Ras mutation status is indicated behind the name: wild-type (−/−); heterozygous (−/+); homozygous (+/+); N*: N-Ras mutation. **C**-**E** Akt isoform-specific kinase activity. **C** Akt isoform-specific in vitro kinase assays were performed with serum-starved cells which were left untreated or treated with EGF (30 ng/ml; 5 min) or IGF1 (10 ng/ml; 5 min). Akt isoforms were precipitated with isoform-specific antibodies and incubated with recombinant GST-GSK3α/β as Akt substrate. To precipitate equal amounts of Akt isoforms the amount of lysate was adjusted according to the expression. Phosphorylation of GST-GSK3α/β and amounts of immunoprecipitated Akt isoforms were detected by Western blotting procedure using antibodies reactive to pGSK3 and Akt1/2/3. Representative blots are shown. **D** The bar graph shows the growth factor-stimulated Akt1–3 activity, as exemplary presented in C. The activity was evaluated by densitometric analyses, related to the amount of precipitated Akt1–3, and normalized to the untreated control cells set to 1. Mean ± SD (*n* = 3–5), one sample t-test, ** *p* ≤ 0.01; * *p* ≤ 0.05 is shown. **E** Representative Western blots of Akt isoform-specific kinase activity assays of serum-starved parental PANC-1 as well as EGFP- and EGFP-K-Ras(V12)-expressing PANC-1 cells. To control for unspecific binding of Akt [Control-IgG] a mouse-IgG2a antibody was used (*n* = 3)

The alterations in Akt isoform expression prompted us to investigate the isoform expression in different pancreatic and lung adenocarcinoma cells with and without oncogenic K-Ras (Fig. 4B and Table S2). The bar graph in Fig. 4A shows the relative mRNA expression of each *AKT* variant related to the expression of the housekeeping gene *RPLP0*. As for example, PANC-1 showed the highest amount of *AKT2* (2^-ΔCq^: 21.53 ± 10.82), whereas all other cells, except for H1299 lung carcinoma cells (2^-ΔCq^: 5.44 ± 0.81), showed a markedly lower expression rate. *AKT1* was expressed at comparable levels in all analyzed cell lines. Although *AKT3* is very weakly expressed in all cell lines, there are considerable differences. H1299 (2^-ΔCq^: 0.003 ± 0.001) showed the highest relative mRNA expression among these cell lines, whereas *AKT3* is barely detectable in COLO 357 (2^-ΔCq^: 0.00003 ± 0.00002) followed by PANC-1 (2^-ΔCq^: 0.0005 ± 0.0004). Statistical analyses were performed for each organ separately, demonstrating that there are no significant differences in *AKT1* and *AKT2* mRNA expression, except of PANC-1 and H1299. *AKT3* is more differentially expressed across the cell lines. To evaluate Akt isoform expression in more detail, nine pancreatic and nine lung adenocarcinoma cell lines were evaluated by Western blot analyses with panAkt and Akt isoform-specific antibodies. Note, when using the panAkt antibody, Akt1 represents the upper band, Akt2 the lower band, whereas Akt3 is only seen in A549 cell lysate as a faint band running in between (Fig. 4B). Therefore, detection of Akt3 was only possible using the Akt3-specific antibody. The Western blots show a more or less comparable expression of Akt1 and Akt2, except for PANC-1 with a marked overexpression of Akt2, shown also in Cheng et al., 1996 [38]. The expression of Akt3 is more divers, with five pancreatic and two lung carcinoma cell lines showing no or hardly detectable protein bands. Densitometric analyses of 3-7 Western blots are shown in Supplement Fig. S2 and demonstrate the differences among the cell lines, with the strong expression of Akt2 in PANC-1, A549, and H1299 cells and the very low expression of Akt3 in BxPC-3, COLO-357, PANC-1 pancreatic, and H2122 and HCC-44 lung carcinoma cells. Interestingly, the mean value of Akt3 protein expression in pancreatic carcinoma cells exhibiting homozygous K-Ras mutation is 1.98 and thus twofold higher than the mean value of 1.01 for wild type/heterozygous mutated cell lines, including Capan-2, which showed inconsistent expression in the four experiments. Thus, pancreatic carcinoma cell lines expressing only mutated and thus active K-Ras showed enhanced Akt3 expression. This association is not obvious in the analyzed lung adenocarcinoma cell lines.

The upregulation of Akt3 by EGFP-K-Ras(V12) demonstrated in Fig. 3A,B raised the question, whether the activity of Akt3 is also altered. As shown in Fig. 1D, treatment of serum-starved PANC-1 cells with growth factors, such as EGF, results in enhanced Akt phosphorylation at Ser473, which indicates Akt activation. Enhanced phosphorylation of Akt by EGF or IGF1 was seen in all analyzed cell lines (data not shown), however, the activated isoform could not be identified by this method. Hence, the activity of each Akt isoform was evaluated in in vitro Akt kinase assays, in which each Akt isoform was precipitated, and its activity was determined by using recombinant GSK3α/β-GST fusion protein as a substrate (Fig. 4C). To compare the kinase activity of the isoforms, equal amounts of each isoform were precipitated by adjusting the amount of lysate used for precipitation, as far as possible. Note that all three isoforms were precipitated from the same lysate preparation for direct comparison. Figure 4C shows representative Western blots of the phosphorylation of GSK3α/β at Ser21/9 (upper blots) and the amount of precipitated Akt isoforms (lower blots) for selected cell lines. All three Akt isoforms can be activated by EGF or by IGF1 in case of MiaPaCa-2. The kinase activity differs with regard to the Akt isoform and the cell line. The activation of Akt1 and Akt2 was hardly detectable in H23 cells but clearly visible in the other cell lines. Most interestingly, although Akt3 is only weakly expressed and hardly detectable even after immunoprecipitation it is more active than Akt1 or Akt2, indicated by the stronger phosphorylation of GSK3 in all analyzed cell lines (Fig. 4C). Moreover, the relative activity of Akt3 in relation to untreated cells, was significantly stimulated in all cell lines (Fig. 4D). Specifically, the relative activity of Akt3 after growth factor treatment was 13.01 ± 2.81, for H23; 21.19 ± 8.10 for H1299; 36.25 ± 10.91 for PANC-1; 9.17 ± 2.66, for K-RasV12 4.1; and 32.09 ± 8.00 for MiaPaCa-2. The EGFP-K-RasV12-expressing PANC-1 cells do not only exhibit an enhanced Akt3 expression (Fig. 3A, B), but also an enhanced Akt3 in vitro kinase activity compared to the parental and EGFP-expressing PANC-1 cells (Fig. 4E). Densitometric analyses of 3–4 independent experiments revealed for EGFP-K-RasV12 4.1 cells a 1.42-fold and for EGFP-K-RasV12 4.4 cells a 1.44-fold increase (*p* ≤ 0.05) compared to EGFP cells.

The expression and activity data demonstrate that expression of oncogenic K-Ras has a substantial impact on regulating Akt isoform equilibrium, especially promoting Akt3 expression and activity.

### Crosstalk of Akt isoforms

**Fig. 5:**
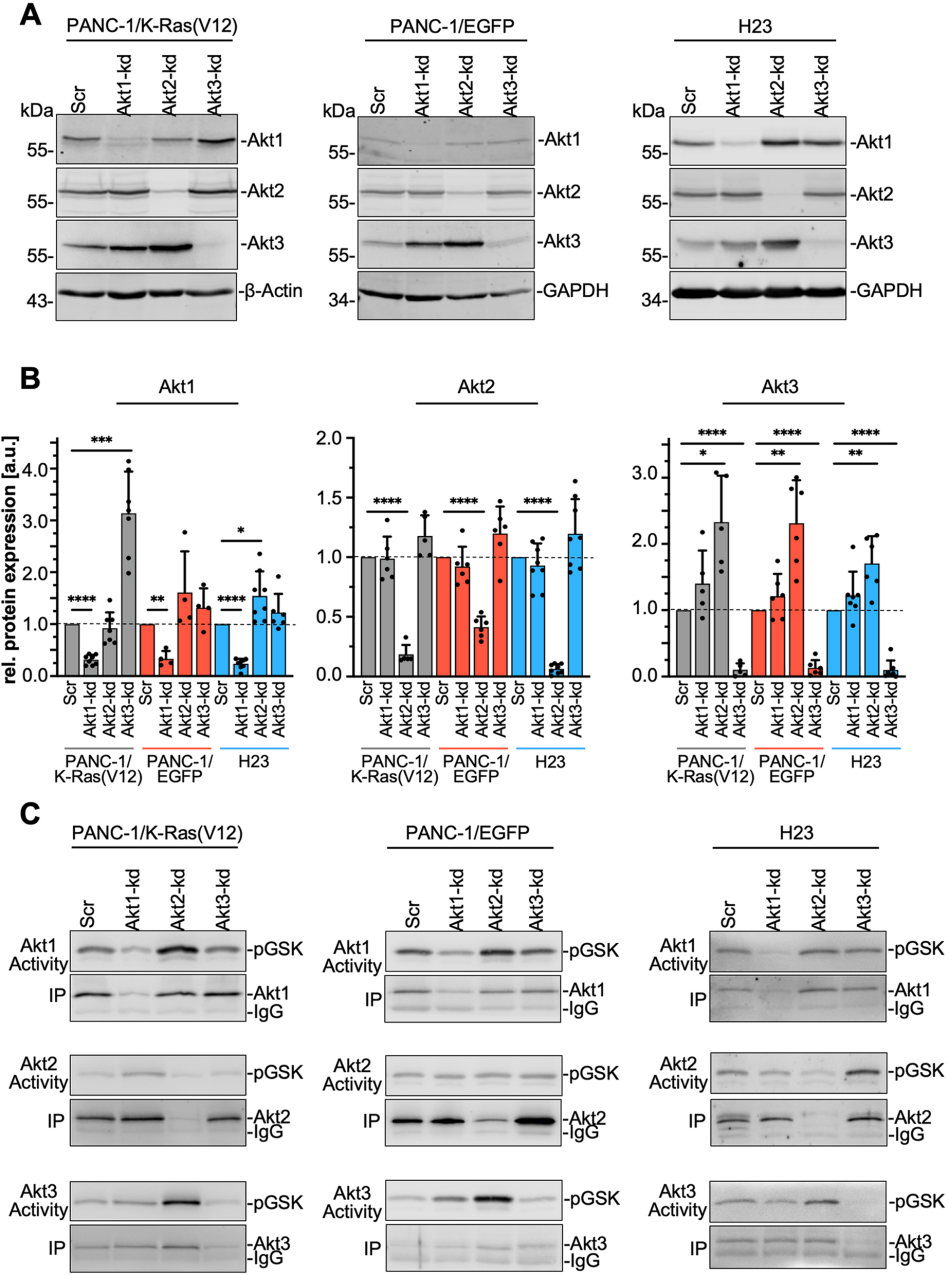
Akt2 depletion upregulates Akt3 expression and activity. **A** Downregulation of individual Akt isoforms was performed by lentiviral transduction in PANC-1/K-Ras(V12) and PANC-1/EGFP pancreatic, as well as H23 lung carcinoma cells using Akt isoform-specific shRNAs. Expression of each Akt isoform was detected by Western blot analysis with Akt isoform-specific antibodies. **B** The bar graphs show the relative Akt isoform expression in relation to GAPDH or β-actin, normalized to the corresponding cell clone transduced with a scrambled shRNA [Scr] set to 1. Mean ± SD (*n* = 5–8, one sample t-test, **** *p* ≤ 0.0001, *** *p* ≤ 0.001, ** *p* ≤ 0.01; * *p* ≤ 0.05) is shown. **C** Akt isoform-specific kinase activity. Akt isoforms were precipitated with isoform-specific antibodies and the precipitate was incubated with recombinant GST-GSK3α/β as Akt substrate. To precipitate equal amounts of Akt isoforms the amount of lysate was adjusted according to the expression, except for the depleted isoform. Phosphorylation of GST-GSK3α/β and amounts of immunoprecipitated Akt isoforms were detected by Western blotting procedure using antibodies reactive to pGSK3 and Akt1/2/3. Representative blots (*n* = 3) are shown

These results prompted us to investigate the functional role of each Akt isoform in more detail by stably downregulating the expression of each Akt isoform using lentiviral transduction with Akt isoform specific shRNA containing vectors. Because of the marked effects of K-Ras(V12) on Akt isoform expression, PANC-1/EGFP-K-Ras(V12), PANC-1/EGFP-expressing cells (Fig. 3), as well as H23 lung carcinoma cells, which express all three Akt isoforms (Fig. 4), were chosen for Akt depletion. Western blot analyses of the Akt knock-down (Akt-kd) clones, presented in Fig. 5A, show that each isoform was successfully downregulated in each cell clone by ≥75%. Interestingly, downregulation of one isoform affected the expression of the other isoforms (Fig. 5A). Specifically, Akt1 was significantly upregulated in PANC-1/K-Ras(V12)/Akt3-kd cells (3.14 ± 0.804) and H23/Akt2-kd cells (1.54 ± 0.47), and markedly but not significantly in EGFP/Akt2-kd cells as compared to the corresponding control cells transduced with a scrambled shRNA (Scr) (Fig. 5B). Moreover, Akt3 expression was significantly increased in all Akt2-kd cell clones by approximately two-fold. The protein amount of Akt2 was not affected by Akt1 or Akt3 depletion. Quantitative PCR analyses revealed a significant depletion of the mRNA of each AKT variant by the specific shRNA. The increase in Akt isoform protein expression was partly mirrored by the upregulation of the corresponding mRNA (Fig. S3A). These results point towards an inhibitory effect of Akt2 on Akt3. In line with the observation that enhanced expression of oncogenic K-Ras downregulates Akt1 and Akt2 expression and upregulates Akt3, a substantial cross-talk of Akt2 with Akt3 could be discovered.

To address the question, whether Akt isoform activities are also affected, the activity of each Akt isoform was analyzed by in vitro Akt kinase assays to directly compare the activity of the precipitated Akt isoforms. Similar amounts of Akt were precipitated by adjusting the amount of the lysate used for immunoprecipitation, except for the corresponding depleted isoform. Representative Western blots in Fig. 5C show the phosphorylation of GSK3α/β as direct indicator for Akt activity (upper blots) and the amount of the precipitated Akt isoform (lower blots). Depletion of Akt2 upregulated the activity of Akt3 by 3.5-fold in PANC-1/K-Ras(V12)/Akt2-kd cells, and 2-fold in PANC-1/EGFP/Akt2-kd and H23/Akt2-kd cells. The activity of Akt2 is significantly upregulated in K-Ras(V12) Akt1-kd cells and markedly in H23 Akt1-kd and Akt3-kd cells. The activity of Akt1 is not significantly affected in the different cell lines and Akt-kd cell clones. The quantification of the densitometric analyses is presented in Fig. S3B. In addition, we evaluated, whether the detection of pAkt^Ser473^ of each isoform is similar to the activity detected in vitro and would thus be sufficient to make assumptions on the protein activity. Each isoform was immunoprecipitated, the amount of pAkt^Ser473^ was analyzed by Western blot procedure and normalized to the amount of the precipitated Akt isoform (Fig. S3C. The phosphorylation analyses resemble the activity data, but there are some differences. Since full Akt activation and activity needs the phosphorylation of both Ser473 and Thr308 [12, 39] the in vitro activity assay accurately resembles the activity of each Akt isoform.

In summary, we demonstrated that oncogenic K-Ras4B interacts with PI3-Kα, activates Akt and differentially modulates Akt isoform expression, with downregulation of Akt1 and Akt2, and upregulation of Akt3. Moreover, a crosstalk of Akt isoforms with regard to expression and activity was discovered. Akt3, although less expressed than Akt1 and Akt2, is the most affected isoform and particularly upregulated and active in Akt2-depleted cells.

### Akt3 regulates E-cadherin and NCAM

**Fig. 6:**
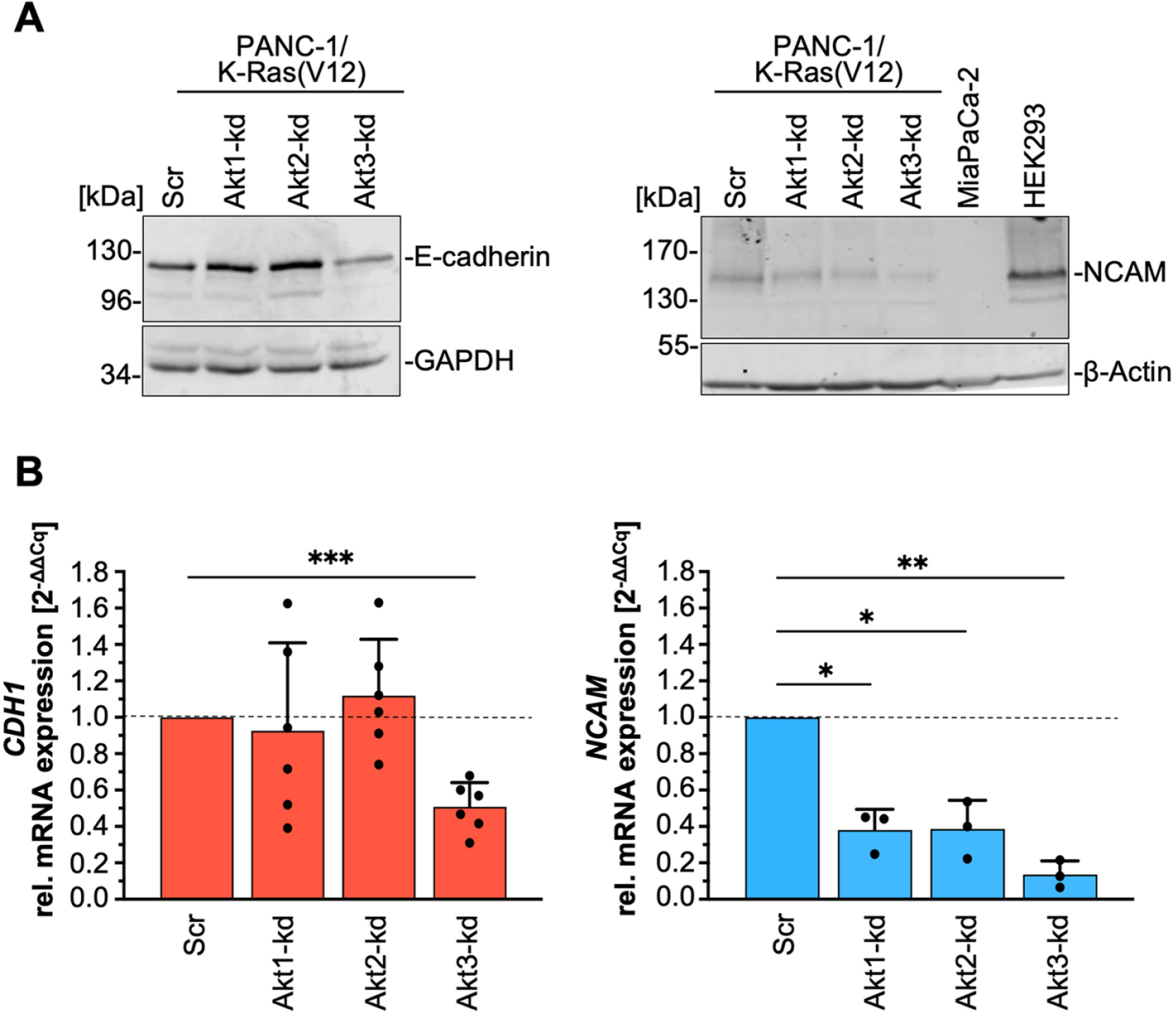
Depletion of Akt3 downregulates E-cadherin and NCAM. **A** The expression of E-cadherin and NCAM was detected in total cell lysates by Western blotting using specific antibodies and GAPDH or β-actin as loading controls. Representative blots of n ≥ 3 independent experiments are shown. For NCAM expression, MiaPaCa-2 and HEK293 cell lysates served as negative and positive control, respectively. **B** The content of the mRNA of *CDH1* and *NCAM* was determined by qRT-PCR analysis. Relative expression was assessed in relation to *RPLP0* and normalized to the expression in the cell clone transduced with a scrambled shRNA [Scr] set to 1. Each sample was analyzed in duplicate. Mean ± SD (*n* = 3–6, one sample t-test, *** *p* ≤ 0.001; ** *p* ≤ 0.01; * *p* ≤ 0.05) is shown

**Fig. 7:**
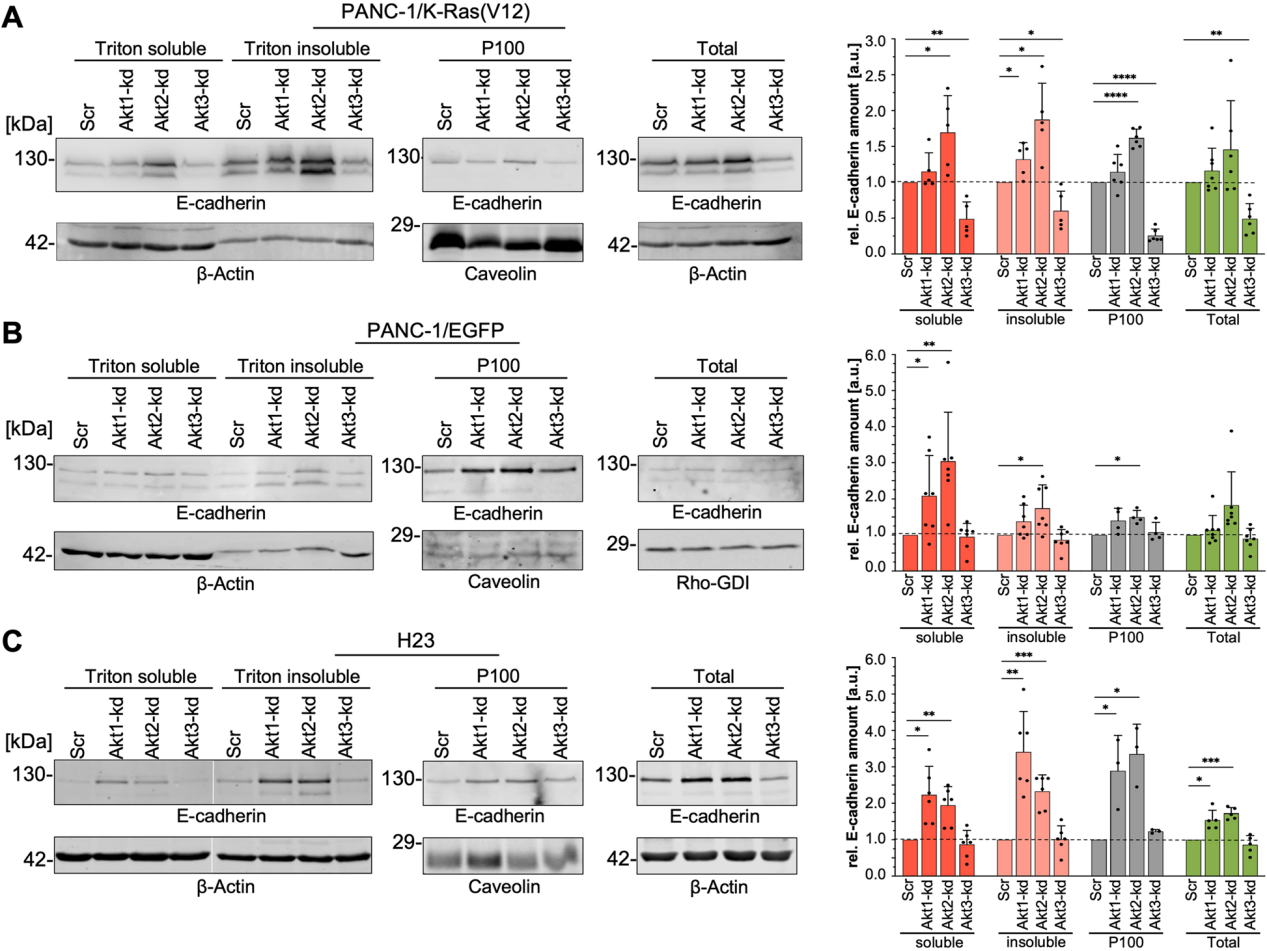
Akt isoforms differentially affect E-cadherin expression and localization. Cellular fractionation experiments were performed to characterize the subcellular distribution of E-cadherin in PANC-1 cells expressing (**A**) EGFP-K-Ras(V12), (**B**) EGFP, and (**C**) in H23 cells with Akt isoform depletion. Triton X-100 fractionation was performed to analyze the association of E-cadherin with the cytoskeleton [Triton insoluble] or as soluble protein [Triton soluble] and crude membrane preparations [P100] for localization within membranes. Total cell lysates were used to detect the total amount of E-cadherin [Total]. Protein lysates (30–50 μg) were analyzed by SDS-PAGE and Western blot procedure using the indicated antibodies. The bar graphs on the right show the relative E-cadherin protein amount estimated by densitometric quantification in each Akt-kd cell clone, in relation to an appropriate control protein, normalized to the corresponding cell clone transduced with a scrambled shRNA [Scr] set to 1. Mean ± SD (*n* = 3–8, one sample t-test, **** *p* ≤ 0.0001; *** *p* ≤ 0.001; ** *p* ≤ 0.01; * *p* ≤ 0.05) is shown

In Schreiber et al., 2008 [22], we have published that enhanced expression of EGFP-K-Ras(V12) resulted in reduced E-cadherin mediated cell-cell adhesion although E-cadherin expression was increased. The concomitant induction of PSA-NCAM interfered with homophilic E-cadherin interaction. Since we have shown here, that Akt3 is a major target of K-Ras(V12), we tested the hypothesis that Akt3 is the regulator of E-cadherin and NCAM in these cells. Since H23 cells did not express NCAM, neither on protein nor on mRNA level (data not shown) they were not included in the NCAM analyses. Western blot analyses of E-cadherin and NCAM expression in total cell lysates (Fig. 6A, left) revealed a 50% reduction of E-cadherin protein amount in the K-Ras(V12)/Akt3-kd cells (0.49 ± 0.21, *n* = 6) compared to the non-target shRNA control cells (Scr) and a slight increase in Akt1-kd and Akt2-kd cells (see also Fig. 7A). This reduction was also seen on mRNA level (Fig. 6B, left) pointing towards a regulatory role of Akt3 on *CDH1* (E-cadherin) transcription or stability. Interestingly, NCAM was also significantly affected by Akt3 depletion. The Western blot (Fig. 6A, right) and the qPCR analyses (Fig. 6B, right) revealed a very strong reduction of NCAM in the PANC-1/K-Ras(V12)/Akt3-kd cells. Specifically, protein expression was suppressed by 75% (0.25 ± 0.08, *n* = 3) and mRNA expression by nearly 90% (0.14 ± 0.04, *n* = 3). Moreover, in contrast to E-cadherin, NCAM expression was also downregulated by Akt1 and Akt2 depletion, although clearly less than by Akt3 depletion.

Downregulation of *CDH1* gene expression can either be mediated via transcriptional repression involving multiple transcription factors or by epigenetic processes such as DNA hypermethylation of cytosine mediated by DNA methyltransferases (DNMTs). The hypomethylating agent 5-Aza-2′-deoxycytidine (5-Azacytidine) is well approved as a DNA methyltransferase inhibitor and hypermethylation of the CpG island in the E-cadherin promoter plays an important role in cancer invasion and dissemination [40, 41]. To test, whether Akt3 might affect *CDH1* promoter methylation, PANC-1/EGFP-K-Ras(V12) Akt3-kd cells were treated with 5-Azacytidine for various time periods and with increasing concentrations to inhibit DMNT-mediated promoter methylation. The effects on E-cadherin as well as N-cadherin expression were analyzed by Western blotting. The results presented in Supplement Fig. S4 demonstrate that neither the amount of E-cadherin nor N-cadherin were affected by 5-Azacytidine treatment. Thus, the regulation of E-cadherin expression by Akt3 is not linked to alteration of the promoter methylation.

Next, we asked if depletion of Akt isoforms also interferes with the subcellular localization and thus function of E-cadherin since only E-cadherin localized in the plasma membrane and associated to the actin cytoskeleton participates in strong cellular adhesion [42]. The amount of E-cadherin associated with the cytoskeleton was determined by Triton X-100 fractionation in PANC-1 cells expressing EGFP-K-Ras(V12) or EGFP, as well as in H23 cells. In addition, membrane-bound E-cadherin was detected in crude membrane preparations generated by cell lysate fractionation by centrifugation at 100.000 x g (Fig. 7A-C). The significant down-regulation of the total amount of E-cadherin by Akt3 depletion in PANC-1/EGFP-K-Ras(V12) cells is also evident in the amount of functional, adhesion relevant E-cadherin present in the P100 membrane-containing fraction and the Triton X-100 insoluble, meaning actin cytoskeleton-associated, protein fraction. Interestingly, neither the EGFP-expressing PANC-1 nor the H23 cell clones with Akt3 knockdown, showed a decrease in E-cadherin content. These results demonstrate that oncogenic K-Ras depends on Akt3 in upregulating functional competent E-cadherin. In contrast, Akt2 depletion upregulated the E-cadherin content in EGFP-K-Ras(V12) as well as EGFP expressing PANC-1 cells and in H23 cells. This increase was especially evident after cellular fractionation: in the Triton insoluble fraction E-cadherin was increased by 1.87 ± 0.51 (*n* = 5) and in the P100 fraction by 1.62 ± 0.13 (*n* = 6) for EGFP-K-Ras(V12)-Akt2-kd cells, by 1.74 ± 0.64 (*n* = 7) and 1.5 ± 0.19 (*n* = 4) in the EGFP-expressing PANC-1 cells, and by 2.33 ± 0.45 (*n* = 6) and 3.35 ± 0.82 (*n* = 3) in H23, respectively, as compared to the control cells. Akt1 depletion resulted in a smaller or similar (H23 cells) increase of E-cadherin (bar graphs in Fig. 7A-C). These data suggest on the one hand, that an increase in oncogenic K-Ras upregulates E-cadherin and NCAM via Akt3 and on the other hand, that Akt1 and especially Akt2 suppress E-cadherin, most likely independent of K-Ras.

### Akt isoforms differentially regulate transcription factors

**Fig. 8:**
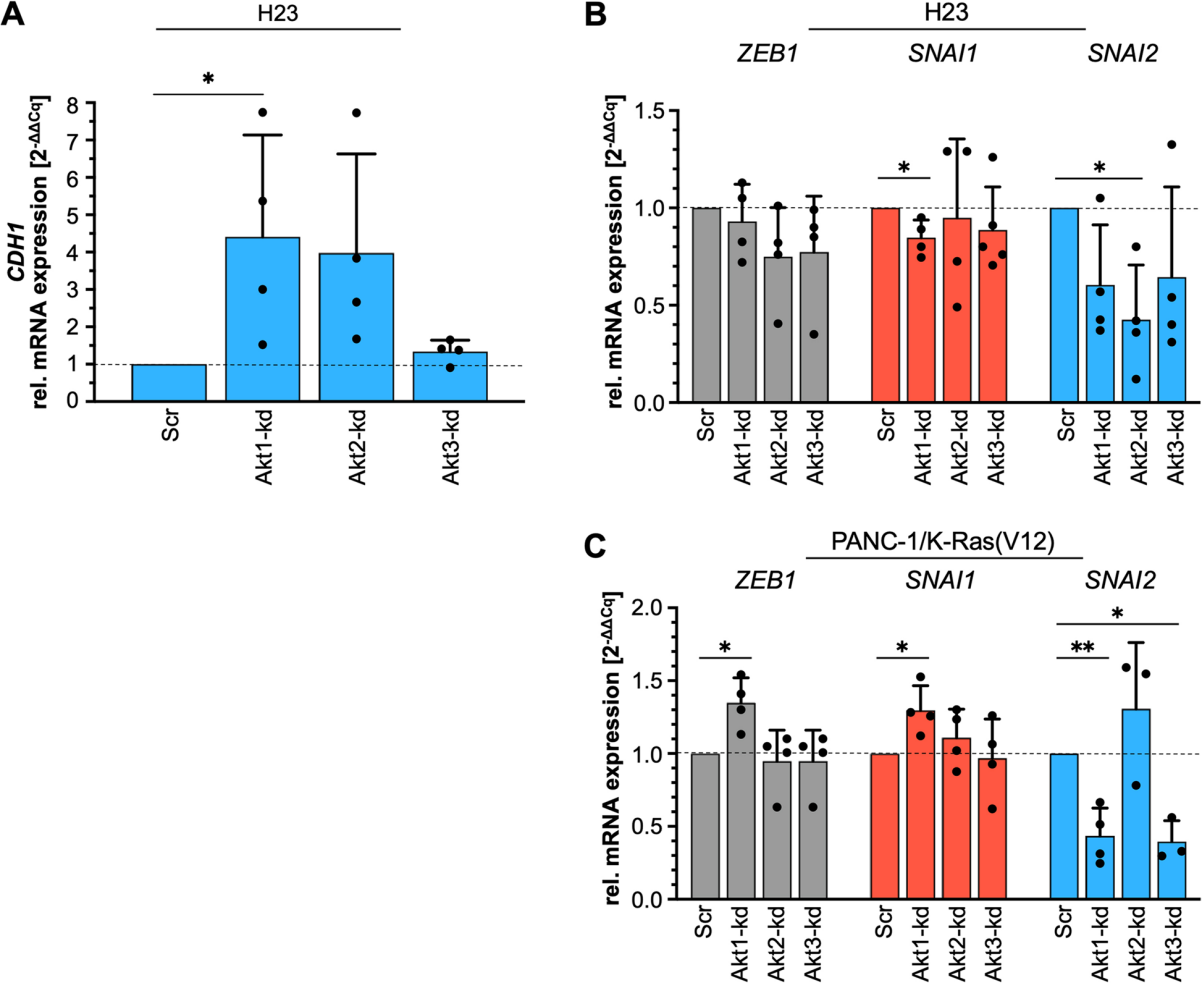
Akt isoforms differentially affect the expression of transcription factors. **A** The mRNA content of *CDH1* is given in H23 Akt-kd clones. Relative expression was assessed in relation to *RPLP0* and to the expression in the cell clone transduced with a scrambled shRNA [Scr] set to 1. Each sample was analyzed in duplicate (*n* = 4). **B**, **C** The mRNA content of  *ZEB1, SNAI1, SNAI2* was determined by qRT-PCR analysis in (**B**) H23 Akt-kd clones and (**C**) PANC-1/EGFP-K-Ras(V12) Akt-kd clones. Relative expression was assessed in relation to *RPLP0* and to the expression in the cell clone transduced with a scrambled shRNA [Scr] set to 1. Each sample was analyzed in duplicate (*n* = 3–5). Mean ± SD (one sample t-test, ** *p* ≤ 0.01; * *p* ≤ 0.05) is shown

The prominent upregulation of E-Cadherin protein content in H23/Akt1-kd and Akt2-kd cells clones (Fig. 7C) was also clearly evident on mRNA level with a 4–5-fold increase of *CDH1*, as depicted in Fig. 8A. To elucidate whether classical E-cadherin regulating transcription factors, namely ZEB1 (*ZEB1*), Snail (*SNAI1*), and Slug (*SNAI2*), are regulated by Akt isoforms and involved in the E-cadherin modulation described above, qPCR analyses were performed. Interestingly, neither the mRNA levels of *ZEB1* nor *SNAI1* were markedly disturbed in H23 Akt-kd cell clones (Fig. 8B). In contrast, *SNAI2*, which shows a low expression rate, was affected by depletion of each of the three Akt isoforms, with the strongest decrease after depletion of Akt2. Comparable effects were detected in A549 lung carcinoma cells (data not shown). In the EGFP-K-Ras(V12)-expressing PANC-1 cells, Akt1 depletion resulted in a slight but significant increase of *ZEB1* and *SNAI1* mRNA (1.35 ± 0.17 and 1.30 ± 0.17, respectively, *n* = 4, *p* ≤ 0,05). Different effects were detected regarding the expression of *SNAI2* mRNA. Depletion of Akt3, led to a significant 60% reduction of *SNAI2,* which correlates with reduced E-cadherin as well as NCAM protein and mRNA expression. Interestingly, Akt1 depletion in EGFP-K-RasV12 expressing PANC-1 cells resulted in a similar reduction of *SNAI2*, whereas Akt2 depletion slightly increased *SNAI2* mRNA. According to these results, EMT-associated transcription factors per se do not seem to be particularly regulated by one specific Akt isoform. But, interfering with Akt expression and Akt isoform equilibrium specifically modulates *SNAI2* expression. In conclusion, especially Akt3 might be involved in the K-Ras(V12) - PI3-Kα - Akt mediated regulation of E-cadherin and NCAM in PANC-1 pancreatic carcinoma cells. Further analyses will be necessary to identify underlying molecular mechanisms regulating E-Cadherin expression in more detail.

## Discussion

In the presented study we have analyzed the effect of oncogenic K-Ras(V12) on PI3-K-Akt signaling and discovered Akt3 as a regulator of E-cadherin and NCAM expression in PANC-1 pancreatic carcinoma cells. Akt1 and Akt2 were characterized as suppressors for E-cadherin expression in PANC-1 as well as H23 lung carcinoma cells.

Oncogenic mutations in K-Ras are highly prevalent in PDACs and occur in 33% of NSCLC [43]. By stable expression of EGFP-K-Ras(V12) in PANC-1 pancreatic carcinoma cells, we previously demonstrated that K-Ras(V12) induced morphological alterations towards a mesenchymal, invasive phenotype, with enhanced cellular motility and invasion, altered actin filament organization, and reduced cell-cell-contact formation [21, 22]. Gene expression analyses revealed reduced expression of certain epithelia markers, such as *CDLN4* (Claudin-4), *KRT8* and *KRT19* (cytokeratin-8 and -19), and increased expression of *CDH2* (N-cadherin). However, E-cadherin was not downregulated in these cells. Interestingly, the Raf-MEK-ERK pathway was not constitutively activated by stably expressed EGFP-K-Ras(V12) and is still sensitive to growth factor activation [21, 44]. Our studies here show that PI3-Kα specifically interacts with K-Ras(V12) and is a bona fide effector of K-Ras (Fig. 1). One of the first descriptions of an interaction between Ras and PI3-K was in 1991, when H-Ras-transformed liver epithelial cells were used in co-immunoprecipitation experiments [45]. The crystal structure of H-Ras with PI3-Kγ enclosed the RBD in p110γ as a 5-stranded β-sheet structure flanked by 2 α-helices [46]. Computational atomic structure modelling exposed a similar structure but also some differences between the K-Ras:p110α and the H-Ras:p110γ interface [47]. Interaction of Ras isoforms with PI3-K results in activation of the kinase and can occur in different ways: First, by RTKs, where the p85 regulatory domain binds to autophosphorylated tyrosine residues of the receptor or via activation of the p110 catalytic domain, which binds to the adaptor complex GRB2/GAP bound to the activated RTK. Second, by RTK-bound adaptor proteins, such as GRB2, which activates SOS and recruits Ras to the complex, followed by direct p110 activation (summarized in [48]). Additionally, binding of Calmodulin to K-Ras can replace the growth factor-activated RTK, which results in interaction with p110α and full activation of PI3-K-Akt [49]. This scenario might explain the stimulatory effect of oncogenic K-Ras(V12) on the activity of PI3-K-Akt independent of growth factors as demonstrated in our study (Fig. 1E). The importance of K-Ras-PI3-K signaling in various cellular processes, such as proliferation, apoptosis, differentiation, and tumorigenesis is generally accepted, although this seems to vary between cell types, tissues, and tumor entities (summarized in [48]). The group of A. Wolfman for example encountered that the growth factor-dependent Akt activation depends solely on K-Ras and cannot be replaced by N-Ras, [21, 50, 51]. Moreover, in genetically engineered mouse models to reproduce *K-RAS* mutant cancers, germline mutation in the RBD of p110α abrogated K-Ras-induced tumor development and decreased tumor burden [52, 53]. These studies are consistent with our findings on the interaction of K-Ras with PI3-Kα and underline the preferential binding of oncogenic K-Ras(V12) to PI3-Kα as compared to H-Ras(V12) and N-Ras(V12).

The expression of EGFP-K-Ras(V12) and its interaction with PI3-Kα resulted in marked activation of Akt not only in PANC-1 pancreatic carcinoma cells but also in Colo-699 and in HEK293 cells, and support the notion, that PI3-Kα-Akt is one of the main effector pathways of K-Ras(V12). Interestingly, we discovered that the stable expression of EGFP-K-Ras(V12) resulted in reduced PI3-K p85α as well as Akt1 and Akt2 expression, but increased Akt3 expression in PANC-1 as well as Colo-699 cancer cells. These effects are mediated either by changes in the *AKT* gene expression or by influencing the mRNA or the protein stability of Akt*,* since both protein and mRNA content were regulated similarly. Previous gene expression analyses in our group underline the downregulation of *AKT2* gene expression in the EGFP-K-Ras(V12)-expressing PANC-1 cells, whereas stable expression of dominant-negative H-Ras increased *AKT2* expression [44].

An upregulation of Akt3 by Ras, especially K-Ras has not been reported thus far but point towards a specific role of this Akt isoform in K-Ras-induced tumorigenesis or malignancy. Although the three Akt isoforms are encoded by separate genes, they share more than 80% amino acids, and overlapping as well as distinct functions have been described in developmental and cellular processes [15, 33]. A comprehensive study by Santi and Lee [54] analyzed the levels of Akt isoform expression in different cell lines. Akt1 and Akt2 expression were very similar in all analyzed cell lines, but Akt3 was less expressed in most of the cell lines, except of HEK293 and HEK293T cells. These data very much mirrored the results obtained in our study using a panel of pancreatic and lung carcinoma cells with or without K-Ras mutation (Fig. 4A,B; Table S2). Akt3 was very weakly expressed, both on mRNA and protein level in all cell lines and was only observable in Western blot analyses using an Akt3-specific antibody. Although, there was no close correlation between Akt3 expression and K-Ras mutation, most of the cell lines with homozygous K-Ras mutation exhibit clearly detectable amounts of Akt3.

In-silico database analyses using cBioPortal (https://www.cbioportal.org) for pancreatic adenocarcinoma (QCMG, Nature 2016) [55] (data not shown) revealed an increased AKT3 expression in KRAS mutated PDAC tumor samples compared to non-mutated samples. Evaluation of 44 PDAC cell lines using The Human Protein Atlas database (https://www.proteinatlas.org/humanproteome/cell+line/Pancreatic+cancer) also revealed that cell lines with high amount of *KRAS* transcripts exhibit elevated *AKT3* transcript levels. Moreover, elevated *AKT3* transcript levels are associated with increased *CHD1* mRNA levels, supporting the finding that upregulation of oncogenic K-Ras increases Akt3 and E-cadherin expression. Molecular mechanisms by which oncogenic K-Ras regulates Akt isoform expression and activity are currently unknown and await further research.

Numerous studies show, that Akt isoforms are differentially involved in tumorigenesis and metastasis. For example, breast cancer cell migration and invasion are promoted by Akt2 but suppressed by Akt1, both in vitro and in vivo [55]. Akt2 can promote migration and invasion by upregulation of β1-integrin in breast and ovarian cancer cells [56] or by increasing the actin-bundling protein palladin [57]. In mouse pancreatic cancer, dual inhibition of K-Ras and Akt2 inhibits cell proliferation and tumor growth [58]. In line with these observations, we showed that inhibition of PI3-K-Akt signaling, either by LY294002 or Akt1/2 siRNA markedly downregulated the motility of PANC-1 and Colo-699 carcinoma cells (Fig. 2). The K-Ras(V12)-induced migration of PANC-1 cells was especially sensitive to Akt2 siRNA (data not shown). This pro-motile effect of Akt2 is not only seen in epithelial tumor cells, but also in mesenchymal stem cells [59]. However, context and cell-type specific effects have to be considered, as seen for example for Akt1, which is pro-migratory in untransformed fibroblasts but anti-migratory in breast cancer cells, or for Akt2 showing the opposite effects in these cell types [60].

Akt3 is the least studied isoform, most likely due to its low expression and more challenging detection, but more and more studies have emerged, characterizing a specific role of Akt3 in cancer, especially breast cancer [19, 61]. According to the Human Protein Atlas (https://www.proteinatlas.org) and the COSMIC Database (https://cancer.sanger.ac.uk/cosmic) Akt3 is upregulated in prostate, colorectal, and lung cancer, as well as certain types of breast cancer cells [15, 62]. Depletion of Akt3 in triple-negative breast cancer (TNBC) cells increased cell migration and metastases formation in mice by upregulation of S100A4 [33]. In addition, downregulation of Akt3 in a bone seeking subline of MDA-MB231 increased bone metastasis which was associated with enhanced activity of HER2 and DDR1/2 described in Hinz et al, 2021 [63]. These data support the importance of Akt3 in tumorigenesis.

Interestingly, we found that depletion of one isoform altered the expression of the other isoforms. Akt3 expression was significantly upregulated after Akt2 depletion in both, the PANC-1 as well as H23 cell clones. Whereas Akt1 expression was only increased in PANC-1 EGFP-K-Ras(V12) Akt3-kd cells. The Akt2-kd cells also exhibit an enhanced Akt3 activity, emphasizing the function of Akt3 (Fig. S2). Akt2 expression was not affected by any of the other depleted isoforms. These data might indicate that loss of Akt2 is compensated by Akt3, a question also discussed by Santi & Lee [54]. They used transient siRNA depletion of each isoform in MDA-MB231 cells and neither the single nor the double knock-down resulted in a different subcellular localization of the remaining isoform [54]. However, this group did not analyze the expression levels of the individual isoforms. Thus, further studies will be needed to answer the question of a compensatory crosstalk on a cellular level.

Besides the crosstalk of the Akt isoforms among each other, a specific role of Akt isoforms in regulating E-cadherin or E-cadherin function, as described here, has not been analyzed before. Our study shows for the first time, that Akt3 supports K-Ras(V12)-induced E-cadherin as well as NCAM expression in PANC-1 pancreatic carcinoma cells (Figs. 6, 7). E-Cadherin and NCAM are expressed in a considerable amount of primary pancreatic carcinoma samples. Molecular mechanisms involved in perturbation of the E-cadherin-mediated cell adhesion, one important step in early stages of metastasis, is of special interest in pancreatic cancer because of its extraordinarily high rates of metastasis. Although enhanced expression of oncogenic EGFP-K-Ras(V12) resulted in increased migration, a more mesenchymal phenotype, as well as a decreased cell-cell adhesion, the expression of E-Cadherin and NCAM was enhanced in these cells [22]. Polysialylated NCAM interacts with E-cadherin and inhibits the E-cadherin-mediated cell-cell adhesion in epithelial, especially pancreatic carcinoma cells. Depletion of Akt3 markedly downregulated both E-cadherin and NCAM expression on protein as well as mRNA level in the K-Ras(V12)-overexpressing PANC-1 cells (Fig. 6), thus classifying Akt3 as a key mediator of oncogenic K-Ras-modulated cell-cell adhesion in pancreatic carcinoma cells. Interestingly, depletion of Akt1 as well as Akt2 had the opposite effect on E-Cadherin expression in K-Ras-mutated PANC-1 pancreatic and H23 lung cells (Figs. 7 and 8), thus pointing towards pro-tumorigenic and EMT-promoting roles for Akt1 and Akt2 in these cells.

For K-Ras-induced lung tumorigenesis in genetically engineered mouse models, Hollander et al. showed that only a deletion of Akt1 inhibited tumor initiation and progression, whereas Akt2 was not essential for tumor formation. Akt3 deletion more than doubled lung tumor size, suggesting that Akt3 may oppose Akt1 in lung tumors. For invasive breast cancer the balance between Akt1 and Akt2 in combination with either high Twist or low miR200 and E-cadherin expression seems to be relevant for the development of an EMT-like phenotype [64, 65]. In the present study, we elucidated whether the classical EMT transcription factors Zeb1/*ZEB1*, Snail/*SNAI1* or Slug/*SNAI2* are also regulated by Akt depletion in H23 or PANC-1 cells expressing K-Ras(V12). The data demonstrated, that only *SNAI2* mRNA was affected, although differentially in the two cell lines, with a downregulation in the H23 lung carcinoma and upregulation in PANC-1 K-Ras(V12)/Akt2-kd clone A contribution of Akt in Slug/SNAI2 regulation is not without presence in the literature. In melanoma cells the matricellular protein SPARC induced Slug expression and E-cadherin downregulation via PI3-K/Akt signaling [66]. Recently, a HER2-Akt-HSF1 Slug axis was described in EMT regulation in breast cancer cells [67]. However, participation of an individual Akt isoform, especially Akt3 has not been addressed.

In conclusion, none of the three transcription factors examined in our study seems to be responsible for the Akt3-mediated regulation of E-cadherin or NCAM on its own. However, since *SNAI2*/Slug is characterized as an E-cadherin repressor, the observed downregulation in H23/Akt1-kd and Akt2-kd clones might account for the enhanced expression of E-cadherin, but there is much left to understand the molecular mechanisms in Akt isoform-mediated E-cadherin regulation.

## Conclusions

This study shows that oncogenic K-Ras(V12) is a key regulator of PI3-Kα-Akt signaling and that the three Akt isoforms crosstalk to each other with regard to expression and activity. Although Akt3 is the least expressed isoform, it is markedly regulated by K-Ras(V12) and is a crucial regulator of carcinoma cell-cell adhesion via modulation of E-cadherin and NCAM expression.

### Supplementary Information

**Additional file 1.**

**Additional file 2.**

**Additional file 3.**

**Additional file 4.**

**Additional file 5.**

**Additional file 6.**

## Data Availability

The datasets used in this study are available from the corresponding author on reasonable request.

## Abbreviations

Akt: Protein kinase B
Akt-kd: Akt knock-down
CBP: Calmodulin binding peptide
Co-IP: Co-immunoprecipitation
ECL: Enhanced chemiluminescence
EGF: Epidermal growth factor
EGFP: Enhanced green fluorescent protein
EMT: Epithelial to mesenchymal transition
ERK: Extracellular-signal regulated kinase
GAP: GTPase-activating protein
GEF: Guanine nucleotide exchange factor
GSK-3α/β: Glycogen synthase kinase 3 α/β
GST: Glutathione S-transferase
HA: Influenza hemagglutinin
HVR: Hypervariable region
IGF-1: Insulin-like growth factor
IgG: Immunoglobulin G
MEK: Mitogen-activated protein kinase kinase
mTOR: mechanistic Target of rapamycin
mTORC2: mechanistic Target of rapamycin (mTOR) complex 2
NCAM: Neural cell adhesion molecule
ns: Non significant
NSCLC: Non-small cell lung cancer
PDAC: Pancreatic ductal adenocarcinoma
PDK1: Phosphoinositide-dependent protein kinase 1
PH: Pleckstrin homology
PI3-K: Phosphatidylinositol 3-kinase
PIP2: 3-Phosphoinositides PI(3,4)P2
PIP3: 3-Phosphoinositides PI(3,4,5)P2
PKB: Protein kinase B
PMSF: Phenylmethylsufonyl fluoride
qRT-PCR: quantitative Reverse transcriptase-polymerase chain reaction
RBD: Ras-binding domain
RTK: Receptor tyrosine kinase
Scr: Scrambled
SDS PAGE: Sodium dodecyl sulfate polyacrylamid gel electrophoresis
*Sf*9: *Spodoptera frugiperda* 9
SOS: Son of sevenless
TAP: Tandem affinity purification
TEV-Protease: tobacco etch virus protease
TF: Transcription factor
