# Supplementary figures and images for: K-Ras(V12) differentially affects the three Akt isoforms in lung and pancreatic carcinoma cells and upregulates E-cadherin and NCAM via Akt3
Source: Cell Commun Signal. 2024 Jan 30;22:85. doi: 10.1186/s12964-024-01484-2 (PMC10826106; doi:10.1186/s12964-024-01484-2)

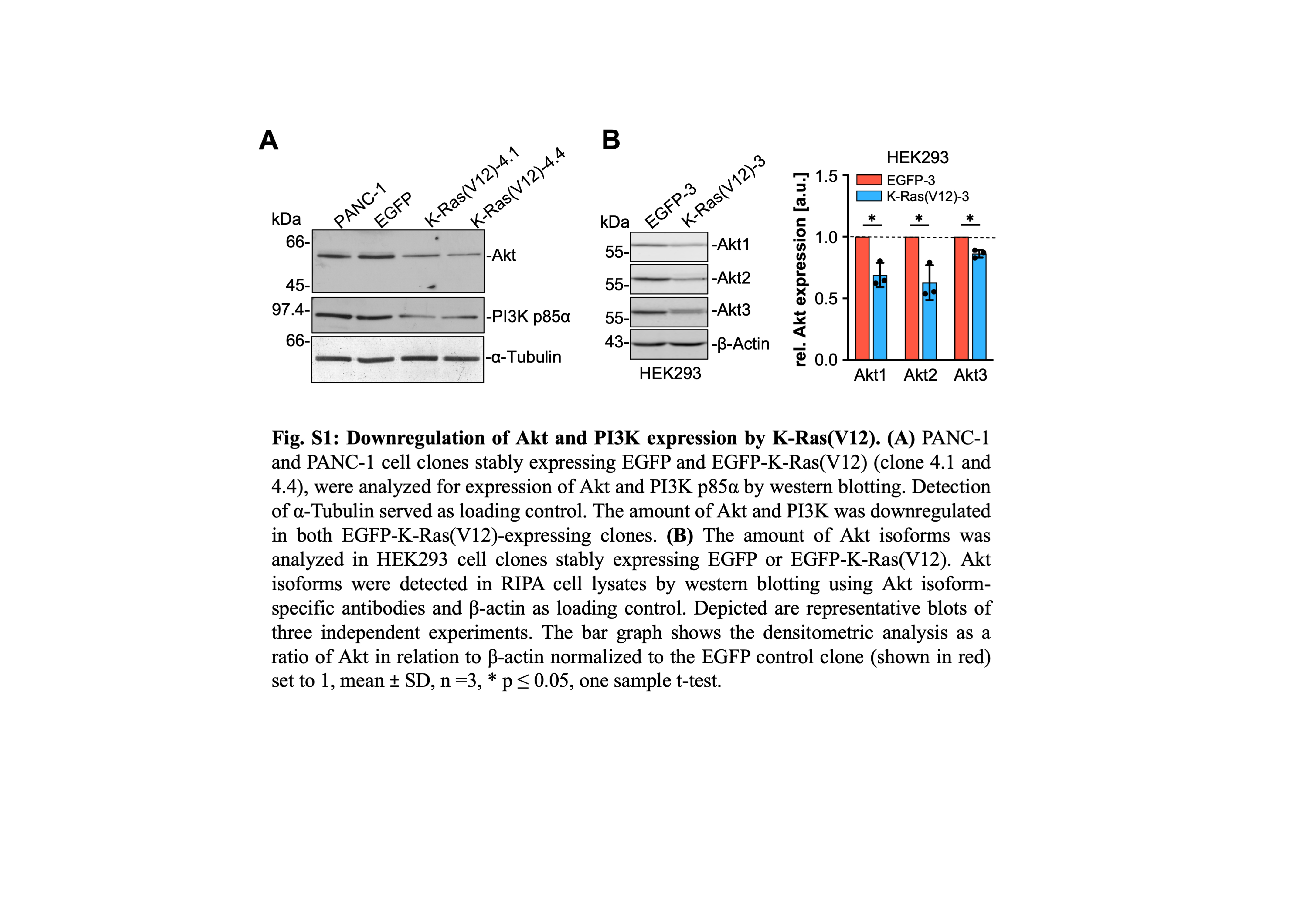

Supplement: Supplementary file 1 — Additional file 1. [file 12964_2024_1484_MOESM1_ESM.jpg]

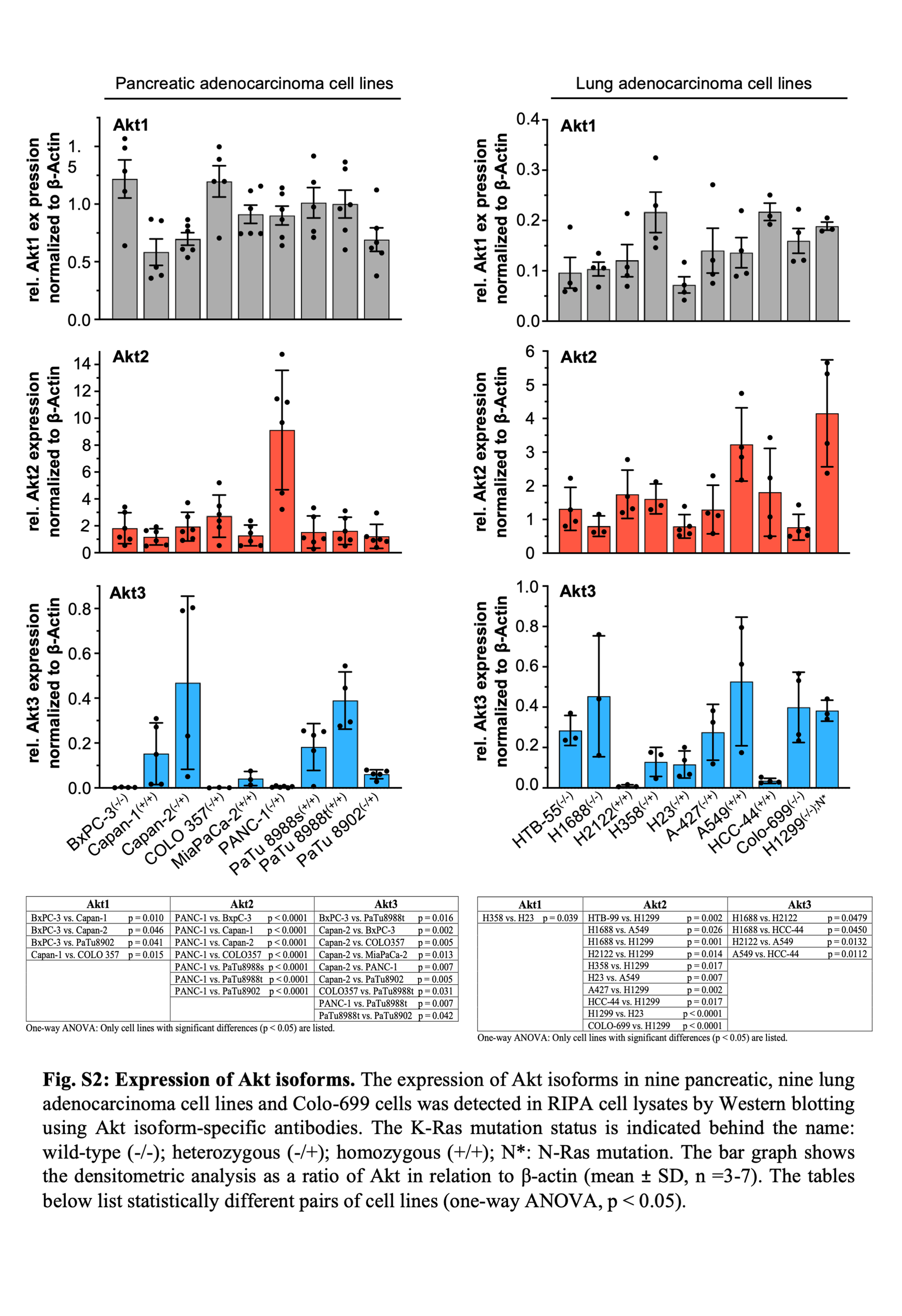

Supplement: Supplementary file 2 — Additional file 2. [file 12964_2024_1484_MOESM2_ESM.jpg]

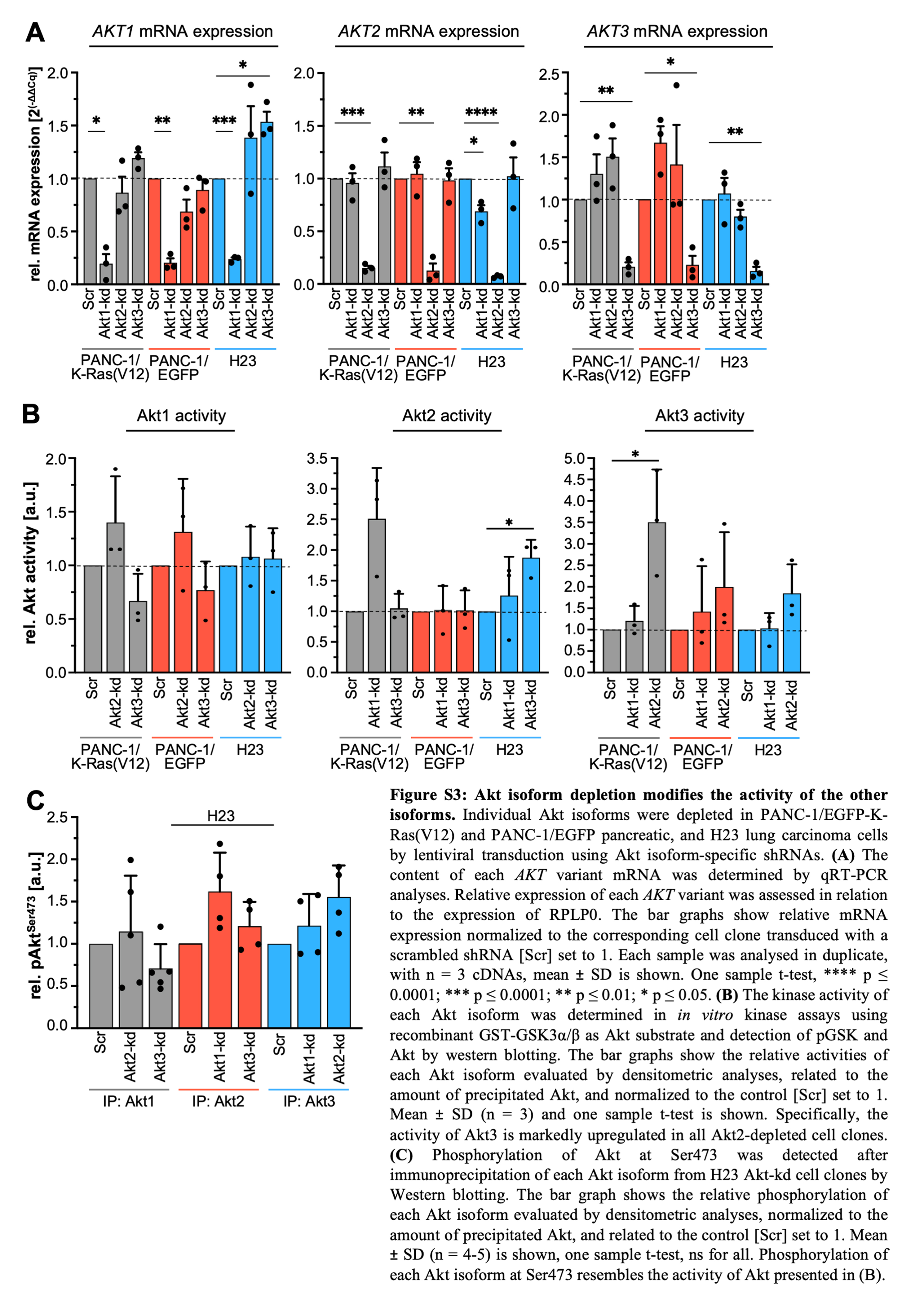

Supplement: Supplementary file 3 — Additional file 3. [file 12964_2024_1484_MOESM3_ESM.jpg]

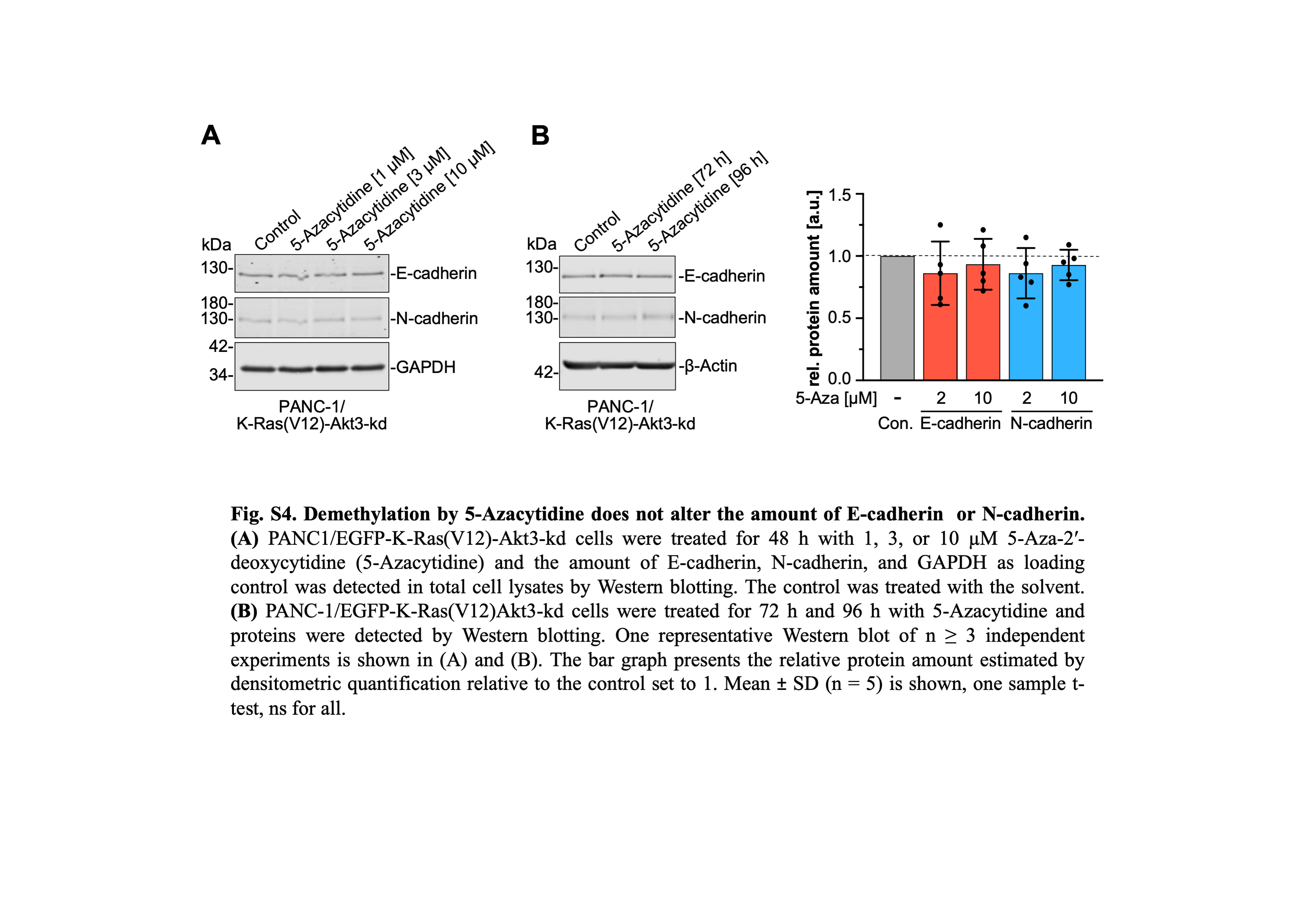

Supplement: Supplementary file 4 — Additional file 4. [file 12964_2024_1484_MOESM4_ESM.jpg]
