# Supplementary material for: K-Ras(V12) differentially affects the three Akt isoforms in lung and pancreatic carcinoma cells and upregulates E-cadherin and NCAM via Akt3
Source: Cell Commun Signal. 2024 Jan 30;22:85. doi: 10.1186/s12964-024-01484-2 (PMC10826106; doi:10.1186/s12964-024-01484-2)
Supplement: Supplementary file 5 — Additional file 5. [file 12964_2024_1484_MOESM5_ESM.pdf]

Table S1: Primary and secondary antibodies

Primary Antibody	Host	Obtained from	Used for Fig.
Akt (pan); #2920	mouse	Cell Signaling Technologies, Leiden, The Netherlands	Fig. 4B-E; Fig. 5C
Akt (pan); #9272	rabbit	Cell Signaling Technologies, Leiden, The Netherlands	Fig. 1C-F; Fig. 2; Fig. S1A
Akt1; #200-301-I51	mouse	Rockland, Limerick, Ireland	Fig. 4; Fig. 5A+B
Akt1 (2H10); #2967	mouse	Cell Signaling Technologies, Leiden, The Netherlands	Fig. 3A; Fig. 4B
Akt1 (C73H10); #2938	rabbit	Cell Signaling Technologies, Leiden, The Netherlands	Fig. 3B; Fig. 5A+B; Fig. S1B
Akt2 (D6G4); #3063	rabbit	Cell Signaling Technologies, Leiden, The Netherlands	Fig. 3A; Fig. 4B
Akt2 (L79B2); #5239	mouse	Cell Signaling Technologies, Leiden, The Netherlands	Fig. 3B; Fig. 5A+B; Fig. S1B
Akt3 (L47B1); #8018	mouse	Cell Signaling Technologies, Leiden, The Netherlands	Fig. 3A+B; Fig. 4B ; Fig. 5A+B; Fig. S1B
Caveolin; #610059	rabbit	BD Transduction Laboratories, Heidelberg, Germany	Fig. 7C
Caveolin-1; #610406	mouse	BD Transduction Laboratories, Heidelberg, Germany	Fig. 7A+B
E-Cadherin; #610182	mouse	BD Transduction Laboratories, Heidelberg, Germany	Fig. 6A; Fig. 7; Fig. S4
ERK2 (C-14); #sc-154	rabbit	Santa Cruz Biotechnology, Heidelberg, Germany	Fig. 2
GAPDH; #sc-25778	rabbit	Santa Cruz Biotechnology, Heidelberg, Germany	Fig. 3A+B; Fig. S4
GAPDH; #60004-1-lg	mouse	ProteinTech, Manchester, UK	Fig. 6A; Fig. 5A+B
GFP (Klon7.1&13.1); #1814460	mouse	Roche Diagnostics, Mannheim, Germany	Fig. 1
N-Cadherin; #610920	mouse	BD Transduction Laboratories, Heidelberg, Germany	Fig. S4
NCAM (123C3); #ab9277-1	mouse	Abcam, Berlin, Germany	Fig. 6A
Phospho-Akt (Ser473); #9271	rabbit	Cell Signaling Technologies, Leiden, The Netherlands	Fig. 1E-F; Fig. 2
Phospho-Akt (Ser473) (D9E) XP; #4060	rabbit	Cell Signaling Technologies, Leiden, The Netherlands	Fig. 4E
Phospho-GSK-3α/β (Ser21/9); #9331	rabbit	Cell Signaling Technologies, Leiden, The Netherlands	Fig. 4C+E
PI3K p58α	rabbit	Marcus Thelen, Bellinzona, CH	Fig. 1A+B; Fig. S1A
RhoGDIα (G-2); #sc-373724	mouse	Santa Cruz Biotechnology, Heidelberg, Germany	Fig. 7B
α-Tubulin; #T5168	mouse	Sigma-Aldrich Chemie GmbH, Taufkirchen, Germany	Fig. S1A
β-Actin ; #AC-15	mouse	Sigma-Aldrich Chemie GmbH, Taufkirchen, Germany	Fig. 4B; Fig. 5A+B Fig. 6A; Fig. 7; Fig. S1B; Fig. S4

Secondary Antibody	Host	Obtained from
CF®680 anti-mouse IgG; #20065-1	goat	Biotium, Fremont, CA, USA
CF®680 anti-rabbit IgG; #20067-1	goat	Biotium, Fremont, CA, USA
IRDye® 800CW anti-mouse IgG; #926-32210	goat	LI-COR, Bad Homburg, Germany
IRDye® 800CW anti-rabbit IgG; #926-32211	goat	LI-COR, Bad Homburg, Germany
Peroxidase-conjugated anti-mouse IgG; #7076	horse	Cell Signaling Technologies, Leiden, The Netherlands
Peroxidase-conjugated anti-rabbit IgG; #7074	goat	Cell Signaling Technologies, Leiden, The Netherlands
