# Supplementary material for: K-Ras(V12) differentially affects the three Akt isoforms in lung and pancreatic carcinoma cells and upregulates E-cadherin and NCAM via Akt3
Source: Cell Commun Signal. 2024 Jan 30;22:85. doi: 10.1186/s12964-024-01484-2 (PMC10826106; doi:10.1186/s12964-024-01484-2)
Supplement: Supplementary file 6 — Additional file 6. [file 12964_2024_1484_MOESM6_ESM.pdf]

Table S2: Eukaryotic cell lines. §Cellosaurus.org; *American tissue culture collection (ATCC); #German collection of microorganisms and cell cultures (DSMZ); NSCLC (Non-small cell lung cancer), SCLC (Small cell lung cancer)

Cell line	Tissue	§Tumor type	§K-Ras Mutation	Reference
AsPC-1	Pancreas	Adenocarcinoma, ascites	p.Gly12Asp/c.35G>A Homozygous	*CRL-1682™
BxPB-3	Pancreas	Adenocarcinoma	Wild type	*CRL 1687™
Capan-1	Pancreas	Adenocarcinoma	p.Gly12Val/c.35G>T Homozygous	#ACC 244
Capan-2	Pancreas	Adenocarcinoma	p.Gly12Val/c.35G>T Heterozygous	#ACC 245
COLO 357	Pancreas	Adenosquamous carcinoma, metastatic site, lymph node	p.Gly12Asp/c.35G>A Unspecified	Morgan et al. (1980) Int J Cancer, 1980
MiaPaCa-2	Pancreas	Adenocarcinoma	p.Gly12Cys/c.34G>T Homozygous	*CRL-1420™
PANC-1	Pancreas	Adenocarcinoma	p.Gly12Asp/c.35G>A Heterozygous	*CRL-1469™
PaTu 8988s	Pancreas	Adenocarcinoma, metastatic site, liver	p.Gly12Val/c.35G>T Homozygous	ACC 179
PaTu 8988t	Pancreas	Adenocarcinoma, metastatic site, liver	p.Gly12Val/c.35G>T Homozygous	ACC 204
PaTu 8902	Pancreas	Adenocarcinoma	p.Gly12Val/c.35G>T Heterozygous	ACC 162
HTB-55 (Calu-3)	Lung NSCLC	Adenocarcinoma, metastatic site, pleural effusion	Wild type	HTB-55
NCI-H1688	Lung SCLC	Small cell lung cancer, metastatic site	Wild type	*CCL-257™
NCI-H2122	Lung NSCLC	Adenocarcinoma, metastatic site, pleural effusion	p.Gly12Cys/c.34G>T Homozygous	*CRL-5985™
NCI-H358	Lung NSCLC	Adenocarcinoma, minimally invasive	p.Gly12Cys/c.34G>T Heterozygous	*CRL 5807
NCI-H23	Lung NSCLC	Adenocarcinoma	p.Gly12Cys/c.34G>T Heterozygous	*CRL-5800™
A-427	Lung NSCLC	Adenocarcinoma	p.Gly12Asp/c.35G>A Heterozygous	HTB-53
A-549	Lung NSCLC	Adenocarcinoma	p.Gly12Ser/c.34G>A Homozygous	*CCL-185™
HCC-44	Lung NSCLC	Adenocarcinoma	p.Gly12Cys/c.34G>T Homozygous	ACC 534
Colo-699**	Lung NSCLC	Adenocarcinoma, metastatic site, pleural effusion	Wild type	ACC 196
NCI-H1299	Lung NSCLC	Lung large cell carcinoma, metastatic site, lymph node	K-Ras: Wild type N-Ras: p.Gln61Lys/c.181C>A Heterozygous	*CRL-5803™
HEK293	Kidney	Embryonal kidney cell line	Wild type	*CRL-1573TM

** Note: While preparing the manuscript the DSMZ discontinued the cell line Colo-699 (ACC 196), because STR analysis demonstrated that their actual cell line is genetically identical to human melanoma cell line RPMI-7932.
